# Critical Review on Magnetically Impelled Arc Butt Welding: Challenges, Perspectives and Industrial Applications

**DOI:** 10.3390/ma16217054

**Published:** 2023-11-06

**Authors:** Mukti Chaturvedi, Arungalai Vendan Subbiah, George Simion, Carmen Catalina Rusu, Elena Scutelnicu

**Affiliations:** 1 Electronics and Communication Department, Dayananda Sagar University, Bangalore 560068, India; cmukti-ece@dsu.edu.in (M.C.); arungalai-ece@dsu.edu.in (A.V.S.); 2 Faculty of Engineering, “Dunarea de Jos” University of Galati, 800008 Galati, Romania; george.simion@ugal.ro (G.S.); carmen.rusu@ugal.ro (C.C.R.)

**Keywords:** MIAB welding, key process parameters, equipment, numerical analysis, joint characterisation, industrial applications

## Abstract

Magnetically Impelled Arc Butt (MIAB) welding is a cutting-edge joining method that replaces the conventional welding procedures such as resistance, friction, flash and butt welding. It is a solid-state process that uses a rotating arc to heat up the butt ends of tubes, being followed by a forging process that completes the joining of the workpieces The magnetic flux density and the current interact to develop the Lorentz force that impels the arc along the faying surfaces. This process is found to produce high tensile strength and defect-free welds in ferrous materials and for this reason, it is predominantly employed in automobile applications for joining metallic tubes. Also, this joining procedure can be applied in the fabrication of boilers, heat exchangers, furnace piping in petrochemical industry and other safety-critical high-pressure machinery parts. The MIAB method has several advantages such as a shorter welding cycle, lower input energy requirement and lower loss of material. Compared to other solid-state welding processes, the MIAB welding has an important advantage in terms of cost-efficient welds with better control and reliability. Moreover, there are researchers who have investigated the joining of non-ferrous dissimilar materials using this welding procedure. The studies have been focused on process parametric analysis that involves optimizing and forecasting the magnetic field and thermal profile distribution. This review article provides competitive insights into various design features, computational methods, tests and material characterization, technical issues and workarounds, as well as automation aspects related to the MIAB-welding process. This work will prove to be a quick reference for researchers, useful to identify the research gaps and conflicting ideas that can be further explored for advancements in joining the similar and dissimilar materials.

## 1. Introduction

**Figure 1:**
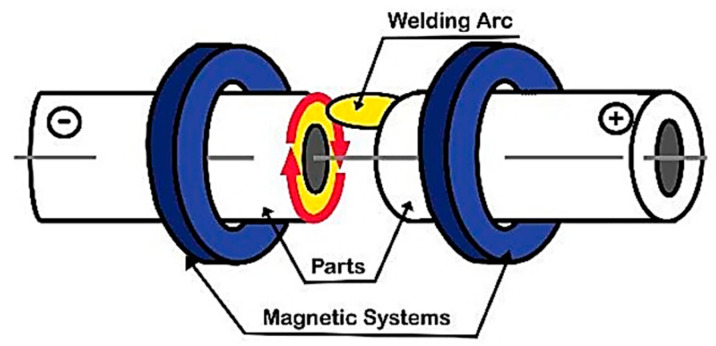
Schematic representation of the MIAB-welding process [9].

Magnetically Impelled Arc Butt (MIAB) welding is a solid-state welding process that uses heat, generated by the rotating arc, to plasticise the parts’ edges which are then fused and joined together by applying high pressure. This unique joining method uses permanent magnets (preferably made of AlNiCo) or electromagnets that generate the magnetic field between the workpieces to be welded (Figure 1). This process can be employed to weld tube to tube or tube to flange, and even to join irregular or non-circular workpieces [1]. The MIAB process is one of the fastest methods to weld tubes by which inclusion- and impurity-free welds are achieved. The joint is completely made in a single cycle, in contrast with other welding processes that require several passes to perform the welded joint. By applying MIAB welding, it is possible to make joints of pipes with an outer diameter from 75 mm up to 450 mm and a wall thickness up to 10–35 mm, in 10–15 s. The power consumption required for the MIAB welding of such tubes is lower in comparison to other solid-state processes [2,3,4,5,6,7,8]. Being an automated process, the results are achieved with a high degree of reliability if the study is replicated. In the MIAB process, the components are not rotated, so the alignment can be maintained without difficulty. This joining method can be employed for welding several alloy steel grades, such as T11, T91, mild steel or combinations of them, and it is also being explored for welding non-ferrous materials, such as Al to Al or Al to Cu [3,4,5,6,7].

A succession of six phases, individualized by process- and parametric-specific changes, is typical to the MIAB-welding process. The tubes are coaxially clamped in the welding head and fixed at distance of 1–3 mm between the faying surfaces. A constant current power source, in a transformer rectifier configuration, is applied to generate the current arc and upset. In the first phase, the tubes are connected to the DC supply, and the arc is ignited by retracting the workpieces. The interaction between the axial component of the arc current and the radial component of the magnetic field, created either by a permanent magnet or an electro-magnet, develops the Lorentz force. This force impels the arc along the peripheral surface of the tubes, marking the second phase of the welding process. Due to the high speed of the arc, as well as the thermal conduction phenomenon, a local uniform heating of the tubes to be joined is developed. However, the arc rotation speed, which is required for joining the workpieces, depends upon the melting point of the base materials. In the third phase, named the arc transitory phase, the heat is generated by the arc rotation on the peripheral surfaces, followed by stable arc rotation and by the arc breaking with a high thermal impact on the surfaces. In the fourth and fifth phases, the edges are pressed with the upset force and, then, due to the heat developed by the electric arc, a localized melting and softening of the adjacent Heat-Affected Zone (HAZ) is obtained. The upset pressure, applied in the last stage on the heated ends, creates a characteristic flash while expelling most of the molten metal along with impurities. Under the pressure exerted on the workpieces, the plasticised zones fuse together, and form a solid phase bond. The clamping arrangement must prevent any axial sliding in the upset phase [10]. Based on experimental observations, the axial magnetic field should be nearly 5–10% of the radial magnetic field vector value and maintained between the parts [11].

Developed in the 1950s at E.O. Paton Electric Welding Institute (EWI), the process gained more importance and relevance in the 1970s and 1980s. Based on the initial experimental results, several acceptances and approvals for application of the MIAB welding in manufacturing particular components have been drawn. These studies focused on improving the arc behaviour control during welding and have enabled further advancements in developing and controlling the process. Gradually, the weld quality was improved and the process has become a cost-effective and eco-friendly joining technique, arousing the interest of specialists for implementing the procedure in manufacturing industry. This process was found to be suitable for welding tubes of thickness <6 mm. It was introduced into commercial applications by Kuka Welding System under the name of Magnet Arc Systems [8]. This welding technique is predominantly employed in the European automotive industry, for a large range of applications, from safety critical components, like axles, pneumatic springs, steering rods, cardan shafts and shock absorbers, to less critical parts, like fuel tank vent pipes.

Researchers worldwide [12,13,14,15,16,17,18] have conducted experimental tests to determine the feasibility of using MIAB welding to join alloy steel tubes. Visual inspection of the weld bead and penetration has been made to analyse the effects of process parameters on the weld geometry. It was reported that incorrect settings of welding current and time cause inadequate heat input and, consequently, inappropriate fusion, whereas an optimal technology generates a qualitative weld with a greater strength than that of the base metal.

Steffen et al. [19] joined different-sized steel tubes and investigated the impact of internal and external magnets, and various power sources on the arc behaviour. To monitor the welding arc, a high-speed video camera and an electronic image converter were employed in the experiments. Significant differences between the behaviour of the arc on the cathode and anode sides of the joint were observed by analysing the images. The arc appeared to move without restriction on the anode side of the joint, but it seemed to be constrained on the cathode side. Moreover, when the arc is forced to move in the presence of a magnetic field, the anode spot is therefore pushed, while the cathode spot is pulled. Moreover, it was observed that the arc always started along the tube edges’ inner diameters (ID). According to the authors, the phenomenon is caused by the arc’ s property to move to the areas that rapidly heat up and to the zones where the demagnetizing effect of the applied magnetic field interact with the induced one that surrounds the arc. It was assumed that the arc length grows as the steel melts along the ID edges. Moreover, because the welding process advances, the arc size increases and the centrifugal forces push it outward and move it toward the Outer Diameter (OD). The authors concluded that the external magnets determined a more controlled rotation of the arc, while the internal magnets accelerated the starting of the welding process. Nentwid et al. [20] compared the impact of magnetic coils, placed internally versus externally, on the arc behaviour during the MIAB welding of tubes. It was found that the edge of the tube closest to the coil always has the highest radial flux density in the weld gap. During the welding process, the magnetic flux density drops significantly along the thickness of the ferromagnetic tube’s wall and affects the arc-starting characteristics.

A bank of batteries, as a potential replacement for DC power supplies, was investigated by Steffen in [19]. Based on the study’s results, it was reported that the arc characteristics have significantly improved. Due to the fact that the source inductance was much lower in comparison with the DC power supply, which had a finite source inductance, the batteries proved to be a much more reliable power source. It is obviously the need for developing and employing power supplies with low source inductance, but it should take into consideration that the batteries are more impractical for such industrial applications. Finally, the authors noticed and demonstrated a strong interdependence between the arc voltage and the tubes gap.

Kachynskyi et al. [21] investigated the MIAB welding of pipes with diameter up to 212 mm, found in various industrial applications and pipeline construction. Implementation of automatic welding methods is expected to achieve reliable welds, increase productivity and reduce the influence of the welder’s abilities on the joints’ quality. For achieving a uniform and stable heating of the faying surfaces, the arc spots of the welding arc need to be proportional to the pipe thickness. In case of larger thickness, the arc active spots have been found to be smaller than the tube cross section, resulting in the short-circuiting phenomenon and, consequently, an unstable welding process. During the process of heating up the metal surfaces, the electric arc was observed to be forced initially to the inside edges and then to the outside edges. Larger pipes thickness determines unstable movement of the arc, leading to the non-uniform heating of material surfaces. For this reason, the inside edges of tubes can be overheated, causing irregularity in weld. The difficulty in welding pipes with walls thicker than 6 mm has limited the applicability of the MIAB welding [22] and that is why further studies on the feasibility of the process for thick tubes are needed. The conditions required for a successful MIAB welding are presented below:

1. The active spots of the rotating arc on the two surfaces to be welded should be nearly the same as the weld thickness. 2. The gap asymmetry and unevenness between the faying surfaces should be less than 0.7 mm.

Based on the above considerations, the scientific approaches which have been conducted with the aim to advance in this process have focused on the control of the arc movement pattern along and across the welding line. For welding thick tubes (thickness > 6 mm), the control mechanism has taken into consideration the radial component of the arc current and the axial component of the magnetic field necessary for initiating the arc movement along the external edges. After the required temperature was reached, the welding current values and the forces were modified, determining the arc to scan the faying surfaces, and then to pass to the upset phase of the parts. This arc control method will completely cover the thick-walled tube cross-section, also ensuring a uniform heating and efficient welding even for the thick-walled hollow and solid parts [11,22].

The continuous development of the process led to an extended range of products, in terms of various geometries of tubes and rods, that can be manufactured by MIAB welding. Because no filler material and shielding gas are required to join the parts, this method is designed to be less expensive, fast, highly productive, with great potential of process mechanisation and automation [21]. However, despite the high speed and reduced cost, several limitations of the process related to the wall thickness, tube diameter, and parts’ shape have to be mentioned. Another limitation of the process comes from the arc (of finite width) that is initially rotating on the inner edge and then predominantly on the outer edge of the tube faces. As a predictable result, uneven heating and consequent poor-quality welds are obtained. This radial movement of the arc path occurs no matter the wall thickness, but becomes less consistent when the wall thickness increases above 6 mm [11,22]. Nevertheless, extensive research work is needed for establishing an optimal parametric window to achieve good welds and for increasing the process adaptability level in the industry. It is obvious that, before initiating any new research on this complex welding process, it is necessary to understand the limitations and the assumptions linked to the MIAB welding. Updating information on this topic is facilitated by this consistent and critical review that highlights the research gaps to be attended and the possible investigation directions to attain solutions that would support a larger implementation and adaptability of the process in the manufacturing industry.

## 2. Key Factors in the MIAB-Welding Process

### 2.1. Magnetic Flux Distribution

**Figure 2:**
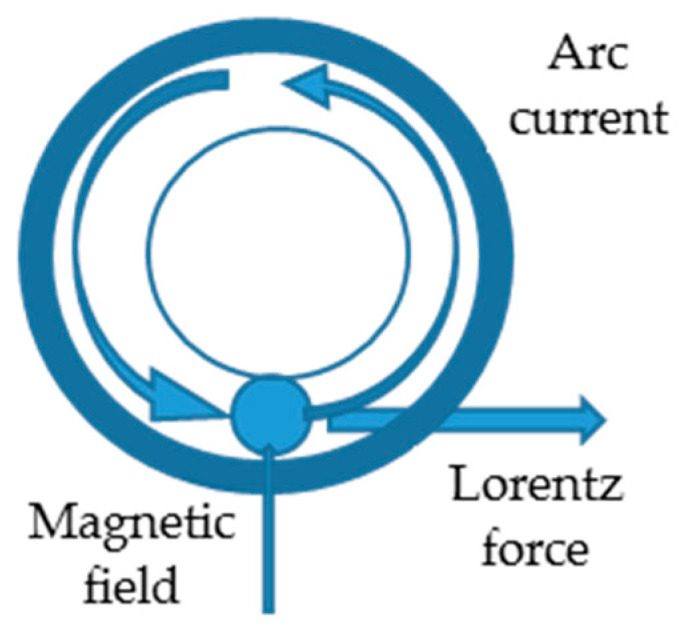
Illustration of magnetic flux, current vectors, and Lorentz force.

The magnetic flux of required density is generated between the tubes by using permanent magnets or electromagnets and, further, the interaction between the axial current and the radial magnetic field develops and controls the Lorentz force that impels the arc along the faying surfaces (Figure 2).

The Lorentz force, created by the interaction between the arc current (*I_a_*) and the magnetic field (*B_r_*), is expressed by the equation

(1) $$F_{BR}=k\;\cdot \;I_{a}\;\cdot \;B_{r},$$

where *k* is a constant that mainly depends upon the gap between the pipes ends and surface preparation.

The pre-programmed control of the arc current determines the arc to reach a velocity of nearly 200 m/s, heating the pipe uniformly. For the MIAB-welding process, the anode and the cathode active spots of the welding arc should be comparable with the wall thickness of the components. The appropriate value of the magnetic field ensures steady arcing between the contact surfaces of parts to be welded. In the conventional MIAB process, after heating, 70% of the faying surface forms an overheated structure on the tube internal edges. This overheated area resists deformability, to the detriment of the weld strength.

**Figure 3:**
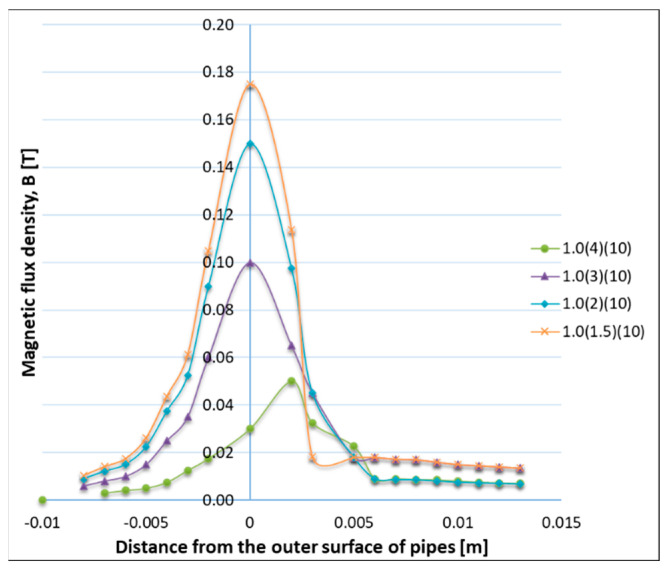
Magnetic flux density versus distance from the tubes outer surface for different coil positions distances and constant current of 1.0 A [12].

**Figure 4:**
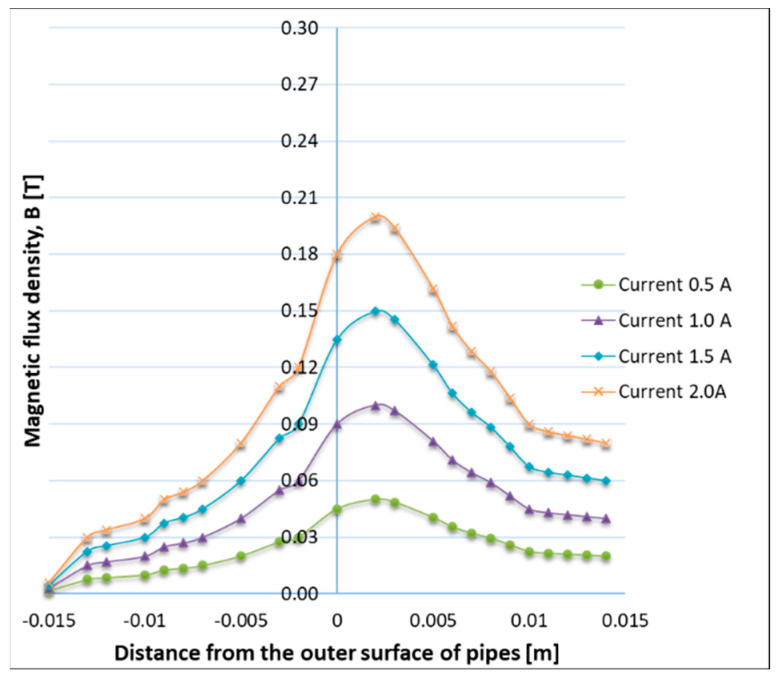
Magnetic flux density versus distance from the tubes outer surface for different current values. Reproduced from [13], Multidiscipline Modeling in Materials and Structures, with permission from Emerald Group Publishing Limited, 2009.

Based on the 3D non-linear finite element electromagnetic analysis (FEA), Vendan, S.A. et al. [12] have investigated the radial magnetic flux distribution, generated in the space between the tubes, by modifying the gap size and the coil position. It was found that the magnetic flux concentration is higher in the space between the tubes when the gap size is minimum, and its effect reduces along the length of the pipe, from one edge to another (Figure 3). The simulation results were experimentally validated by measuring with a Gauss Meter device the radial magnetic flux density at various positions from the pipe surface. The experimental results confirmed the strong interconnection between the magnetic field distribution, on the one hand, and the gap size and coil position, on the other hand. In a similar FEA, Vendan S.A. et al. [13] investigated the magnetic flux distribution for a range of electromagnet-exciting currents and have obtained the flux density variation (Figure 4), by maintaining the tube gap and the coil position constant. The experimental trials, performed to validate the numerical results, were carried out on T11-grade tubes (Cr alloy steels). The authors found that the numerical model can precisely predict the magnetic flux density for a range of exciting currents [13].

New strategies for achieving uniform heating of surfaces in contact with tubes, whose thickness is beyond 6 mm, are worked upon. The active arc spot size in the conventional method, which suits the pipe thicknesses of less than 6 mm, is smaller than the weld cross-sectional area of thick tubes. The proposed system for thick-walled tubes involves the interaction of radial current and axial external magnetic field that induces a force which determines the arc to be displaced to the outer edges of the tubes. The external edge of the tubes is the area where maximum field induction occurs and imparts a higher arc rotation speed. The increased emission of heat provides uniform heating of the faying surfaces. When the level of required heating is reached, the current and the forces acting on the arc determine whether the welding arc is to scan along the ends surface before the upset pressure is applied. A uniform and concentrated heating on the tubes’ perimeter is achieved. An overheated area, representing 30% of the weld area from the external edge, is developed and, then, during the upsetting process, it is removed.

### 2.2. Arc Rotation

**Figure 5:**
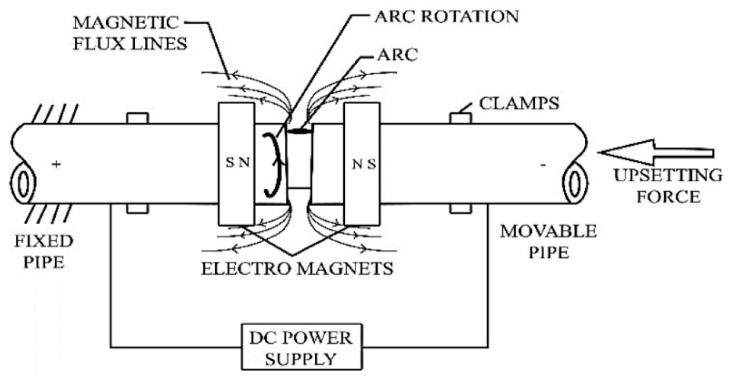
Schematic diagram of MIAB-welding process [16].

Due to the presence of the external magnetic field that is generated with permanent magnets or electromagnets, the welding arc starts to rotate in the gap. On the other hand, the magnetic flux induced in the two tubes to be welded is of the same magnetic polarity on the tubes’ edges (Figure 5).

To provide an explanation in terms of arc movement, researchers [23] used a magnetic transformation model. This model showed a variation of the spontaneous magnetic field produced by the arc over the faying surface thickness. Because of the difference between the inner and outer magnetic fields, geometrical forms, and material properties, a magnetic blow effect is developed. The higher magnetic flux density at the outer edge causes the blowing of the arc towards the inner edge. The arc’s movement from the inner to the outer surface is determined by the decreased magnetic effect in the pipe end. Additionally, because the temperature continues to increase, the magnetic field at the pipe’s inner edge is becoming nearly negligible at the magnetism transition point of nearly 770 °C. Consequently, the pipe is acting similarly to a non-magnetic material and that causes the magnetic flux in the spontaneous magnetic field of the arc to become stronger at the inner edge. This difference in magnetic field causes a magnetic blow on the arc that determines the arc’s movement to the outer edge. Several scientists have stated that due to the presence of oxides, the arc rotates initially on the inner surface, but other researchers have not agreed and not supported this explanation [11].

For tubes thicker than 1.8 mm [11,22], firstly the arc slowly rotates at the inner surface, then, as the temperature rises, the speed of the arc gradually increases, as it moves to the outer edge. The initial low speed is caused by the uneven temperature distribution on the tubes’ ends that may lead to deterioration of the joint quality. The low speed is also attributed to the low flux density of the radial magnetic field at the inner edge. However, in relatively thin pipes of 1.4 mm, the initial speed of the arc rotation has a high value, as the arc covers the entire pipe surface, and the arc flux density is also high at the outer edge. The arc bridge is comparable to the pipe thickness and completely covers the wall. It was also observed that the arc initiation at the inner edge is not related to either the starting position of the arc or to the magnetic field of the exciting coil.

Sato et al. [23] recommended a method for in situ monitoring of the arc rotation velocity and the arc position during MIAB welding. They determined the rotational speed using a phototransistor and an oscilloscope. The arc’s rotation position can be identified by measuring the voltage difference on the outside and inside surfaces of the arc or by interrupting the process and observing the melted zone traces on the weld surface. Using steel, aluminium, and copper alloy pipes, the authors performed an experimental study, in order to confirm the theory of magnetic blow that causes the arc to be pushed to the inner edges of the tubes. In non-magnetic materials, Al or Cu, the magnetic field of the arc is stronger at the pipe’s inner edge, since there is no absorption of the magnetic lines by the tubes. However, in the case of ferrous materials welding, the magnetic field produced by the arc is lower at the inner edge of the pipe. On the inner edge, the magnetic field of the arc is absorbed by the steel tube, thus the stronger magnetic field developed on the external edge blows the arc to the inner surface. The authors noticed that the difference between the flux density values is higher for thicker pipes. Moreover, they linked the arc quenching and the loss of metal magnetic properties in the vicinity of the tubes space to exceeding the Curie point.

To determine the arc speed and the angle, Taneko et al. [24] employed a high-speed video camera, an oscilloscope, and a voltage detector that were positioned in various locations inside a carbon steel pipe. The aim of the study was to investigate the connection between the arc speed, arc angle, and the location where the power supply was applied. Due to the arc blow effect and tube’s low electrical resistance, the current in the arc increases as it moves closer to the power supply position and that enhances the magnetic blow effect while the welding arc is slowing down. It was noticed that the arc speeds up as it moves away from the power source. Based on these observations, the authors concluded that to sustain a steadily moving arc, it is critical to have multiple uniform contact sites on the tube. Moreover, they suggested some local changes in terms of the magnetic field direction and strength to be made for developing a stable arc. Also, the authors explain the normal magnetic field distribution, how it is developed and how it affects the welding arc behaviour, which is useful and valuable information for the specialists interested in this joining technique.

### 2.3. Arc Velocity

The arc is considered to have a virtual mass m, in kg, acquired by the molten steel that is assumed to be travelling with the arc, driven by the force *F*, through air drag resistance *kv*, in kg·m/s^2^, which is proportional with the velocity *v*, in m/s, at a right angle to its motion direction. The mathematical relation of motion for this system is given by Equation (2) [24]:

(2) $$F-kv=m\;\cdot \;\frac{dv}{dt},$$

Considering the initial condition *v* = 0, when *t* = 0, and the final condition *v* as infinite, the solution of Equation (2) can be written as follows:

(3) $$v=v_{\infty }\left(1-e^{-\gamma t}\right):where\;\gamma =\frac{k}{m},v_{\infty }=\frac{F}{k}and\;thus,\;V_{\infty }=\frac{F}{\gamma m},$$

The authors [24] used the velocity equation to plot the arc velocity versus time curve for *v*_∞_ = 58 m/s and *γ* = 2 s and they reported three main levels of variation:

1. The arc initiates with a low velocity and increases gradually to 10 m/s.
2. The arc reaches the high velocity of nearly 55 m/s with negligible fluctuation.
3. The arc has severe velocity fluctuations, depending on the current values and flux density, roughly in the range of 55 to 200 m/s.

The arc rotates steadily along the faying surfaces in the levels 1 and 2 and moves around the pipe end surfaces with irregular deceleration and acceleration in the level 3. In the low-velocity region, the arc is observed to be directed towards the centre of the pipe, but this is not the case in the region of high rotation velocity, where the fumes appeared to be dragged by the arc [24]. In the time range where the arc velocity fluctuates, the fumes are not seen anymore, but the formation of spatters was noticed. Frequently, whenever the short circuit occurs, the arc, that has an irregular movement, is interrupted. Thus, the bridge tends to rotate gently, and as it becomes broken, the arc is reignited. The time required to change the arc velocity from low (about 8 m/s) to high value (55 m/s) is inversely proportional to the welding current. Under the influence of the magnetic field, it is observed that the arc velocity reaches around 210 m/s, and then it is gradually dropping.

The velocity of the arc movement between a tube and a plate was examined by Yatsenko et al. using galvanometers and photoelectric cells [25]. The researchers specifically assessed the impact of the arc gap fluctuations and the welding parameter modifications on the arc speed. It was found that the welding current, magnetic field strength, arc gap size and temperature of metals being welded have a strong influence on the arc velocity. Also, the scale and oxides existent on the faying surfaces of the joint affect the arc mobility and velocity. Removing the scale from the plate and tube ends, an increase in mobility of the arc was noticed. The analysis revealed some differences between the anode (tube side) and the cathode (plate side) spots. Particularly, when metal vapours were developed, the anode spot was disrupted, and an evident jump-like movement was visible. It was suggested that the anode spot area growth determines the increase in temperature, and when further increasing the workpiece temperature, the arc speed increases too. Due to the decrease in the current density and rigidity of the arc plasma, the arc column can be more easily modified by the magnetic field. Furthermore, it was noticed that higher distortion encourages the growth of new anode spots and, consequently, the arc speed increases. On the tube’s ends, a layer of molten metal quickly creates a wave bridge, shortening the arc length by reducing the gap. Further, when the arc length decreases, the arc plasma becomes stiffer, making it more challenging for the applied magnetic field to move it. This explains why the arc velocity reduces after the initial peak. As the gap between the tubes ends widens, the arc velocity increases again. This is a result of the reduction in arc rigidity which is described above. As the components are heated further, the arc velocity increases, partially because the electromagnetic forces are driving out more molten material. Due to the high arc speed that produces large amounts of molten metal which form bridges and cause the instability of the arc, a final decrease in the arc velocity was observed.

### 2.4. Heat Transfer during MIAB-Welding Process

The arc rotation speed is much faster than the heat dissipation rate in the metallic parts and, consequently, a uniform heating of the faying surfaces is achieved. For the finite element modelling (FEM), the internal heat generation was considered as ellipsoidal internal volumetric heat generation. Due to the rotating arc, at the middle of the pipe, the heat input is given by Equation (4) [26]:

(4) $$\dot{Q}=\frac{3{\dot{Q}}_{T}}{2\pi^{2}R_{o}ab}e^{\left(\frac{-3z^{2}}{a^{2}}-\frac{3r^{2}}{b^{2}}\right)},$$

where *a* and *b* represent the welding arc size, in radial and axial directions, being assumed to be around 1 mm on each direction.

The arc rotation speed is also observed to be related to the variation of the arc position, as a function of time, along the wall thickness.

(5) $${\dot{Q}}_{T}=\eta \;\cdot \;V\;\cdot \;I,$$

where *Q_T_* is the heat input; *V*, arc voltage (82 V); *I*, welding current (6 A); and *η*, heat transfer efficiency (70%).

Combining the convective and radiative heat transfer, the following relationships can be written:

(6) h=0.0668·T for 0<T〈500 and h=0.232·T−82.1 for T 〉500

The arc can be considered a constant heat source, and the temperature, at any time *t* and distance *y* from the arc initiation, can be described by Equation (7) [9]:

(7) $$T\left(y,t\right)=\frac{qy}{2\lambda \pi }\left\{\left[\frac{\sqrt{4at}}{y}\;exp\left(-\frac{y^{2}}{4at}\right)-\sqrt{\pi }\;\left[1-\phi \left(\frac{y}{\sqrt{4at}}\right)\right]\right]\right\}$$

**Figure 6:**
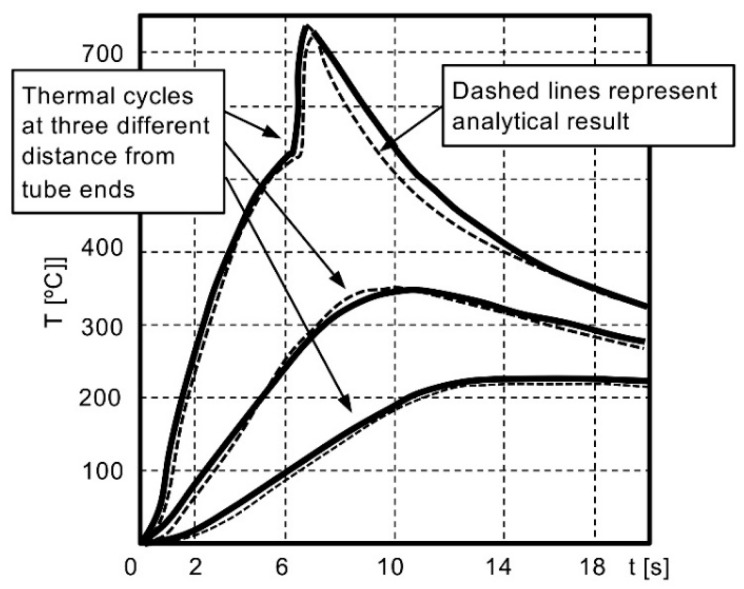
Profile of predicted (dashed line) and experimental (solid line) heat flow [26].

To obtain a quality weld, the temperature *T* and the distance *y* should be correlated. Thus, for a given temperature and distance, an optimum relationship between the heat input and arc rotation time can be established and, further, the width of the HAZ can be determined by applying it. Due to the different levels of current, measured at the start of the process, the heat input cannot be considered constant [9]. Based on heat flow theory, mathematical equations for predicting the temperature versus time have been developed and then the analytical results have been compared with the thermal cycles experimentally recorded at various distances from the tube ends, as shown in Figure 6 [26].

Loebner et al. [27] provided a brief overview and reported their substantial experimental attempts. It should be mentioned that no significant research on the critical topic of heat flow, developed during the MIAB welding, was published so far. In order to reduce the thickness of the liquid molten metal and to obtain quality welds, a balance between the localised heating made by the arc and the heat transferred by conduction into the workpiece is required. An adequate heating reduces the yield strength of the adjacent solid metal and promotes the creation of liquid layers that makes the upsetting of part ends easier. Kachynskyi et al. [21] studied the influence of the metal localised melting during upsetting phase and the effect on the weld formation.

**Figure 7:**
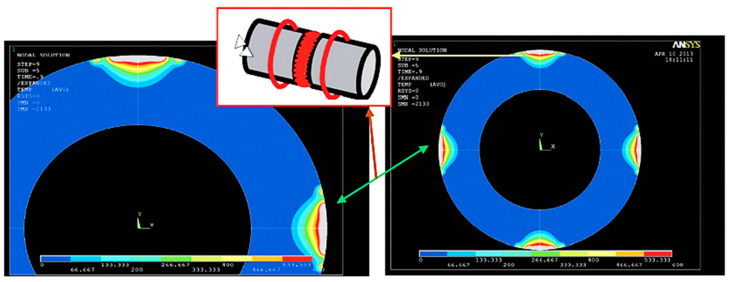
Temperature distribution for high-speed arc or analogous multi-arc system. Reproduced from [14], International Journal of Applied Electromagnetics and Mechanics, with permission from IOS Press, 2014.

Using the Finite Element Method (FEM) to predict the temperature distribution along the tube’s periphery and thickness, Arungalai Vendan, S et al. [14] performed a nonlinear transient thermal analysis during the arc movement in the welding process. Considering the radial, circumferential and axial directions, the heat transfer is assumed as three-dimensional. In the weld reinforcement, the temperature reaches approximately 450 °C, and the weld line indicates a higher temperature gradient. The researchers reported that the arc’s high speed, nearly 250 m/s, can be modelled as multiple arcs at different locations. This model was used to analyse the zigzag and the circumferential arc movement along the joint surfaces. Based on this study, the temperature distribution with arc trailing from the outer diameter (OD) to the inner diameter (ID) should be correlated with the required speed. By changing the arc position from ID to OD, during the arcing phase, it was evident that the modification of temperature distribution versus time was determined by the modification of the magnetic field. These results were experimentally validated using IR temperature sensors that monitored the temperature change during the process. For a better accuracy in observing the temperature distribution, the authors extended the study to the four-arc system, also noticing a good weld penetration (Figure 7). Additionally, the magnetic arc blow phenomenon generated during the MIAB process was illustrated.

Sivasankari, R. et al. [15] observed a wide Light Band (LB) zone, along the weld line, in the microstructure of the MIAB-welded carbon steel tubes of 44 mm OD and 4.5 mm thickness, the phenomenon being attributed to the lower current (600 A) applied during upsetting. Incomplete homogenization and upsetting cause chemical heterogeneity that results in decarburization during the phase transformation. A higher arc rotation increases the melting, and, consequently, the widening of the LB zone. Moreover, due to increasing the ferritic structure content in the weld, the LB zone is characterised by lower tensile strength. Welds, made with higher arc rotation and lower current applied during upsetting, have a wider LB zone and may fracture at the interface during the tensile test.

Various scientific articles, cited in this work, focused on the interaction of electromagnetic and mechanical forces, and their impact on the welding process dynamics. Based on the offset conditions, several process aspects were found inadequate. The offset conditions may include changing the magnetic field, heat losses, inappropriate arc position, and velocity. The magnitude of the magnetic field is the main key factor that influences the arc rotation and controls the velocity and its placement in the tube gap. A part of the heat, generated on the peripheral surface of the joint, is transferred by conduction in workpieces and another part is lost by convection and radiation phenomena, but these heat losses are assumed to be negligible in the research. The arc movement in the tube gap, from inner to outer diameter, may cause generation of non-uniform heat that has a crucial role on the quality of welds. As regards the techno-economical evaluation of this process, with respect to the conventional solid-state welding process, no results have been identified in the available literature. It is obvious that new advancements in researching the MIAB-welding process can be achieved only by studying, comparatively, the influence and the modification of process conditions on the joints’ quality.

## 3. Equipment Used in MIAB Welding

The MIAB-welding process begins with the formation of sparks that are determined by the retractation of tubes. The optimum welding current and the gap width ensure the arc formation and the connection between the tube faces. Researchers working in the MIAB welding field have established a list of basic design factors for welding tubular components, such as the qualitative preparation of pipe ends, optimal distribution of the control magnetic field induction (CMF), arc voltage, welding current and time, and rate of shortening the arc gap during the upsetting process [9,10,19,21,22,28].

**Figure 8:**
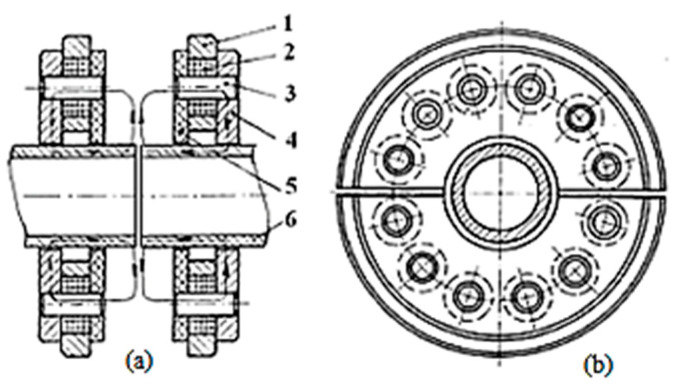
MIAB device design: (**a**) parts of the device (**b**) longitudinal small coils. Reproduced from [29], Annals of “Dunarea de Jos” University of Galati, Fascicle XII, Welding Equipment and Technology, with permission of “Dunarea de Jos” University of Galati, 2001.

A pneumatically operated ROTARC portable device was designed by Georgescu V. et al. to weld pipes (6) up to 30 mm diameter [29]. The researchers aimed to achieve an equipment capable of ensuring the high-speed forging. The magnetic-field-generating system comprises longitudinal small coils that can be opened in half, so that the pipes to be easily manoeuvred (Figure 8). The coils (2) were positioned between the ferrous support (4) and the non-magnetic support (5), shielded by the non-magnetic shells (1). The coil (3) core has the role to minimise the magnetic circuit reluctance and, therefore, to intensify the magnetic field while remaining independent. The pressured air was simultaneously supplied to both pneumatic motors, but the main motor (the vertical chamber) can turn on only when the horizontal motor unlocks it.

**Figure 9:**
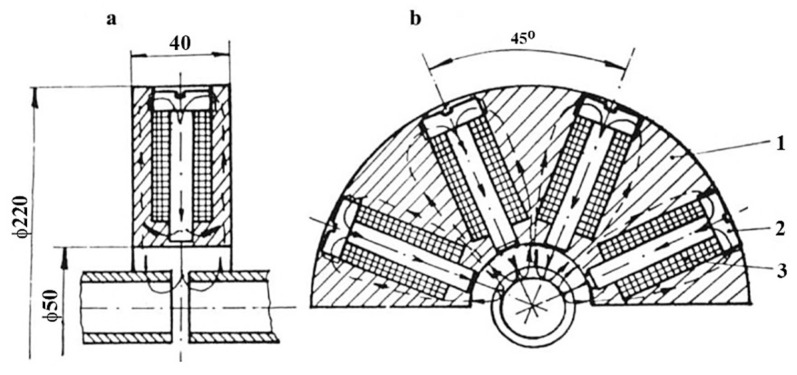
Principle sketch of the transverse magnetizing system: (**a**) superior half-disk (**b**) parts of the device and radial arrangement of coils. Reproduced from [30], Annals of “Dunarea de Jos” University of Galati, Fascicle XII, Welding Equipment and Technology, with permission of “Dunarea de Jos” University of Galati, 2000.

Georgescu, V. and Iordachescu D. developed a Magnetarc welding technology in the Research Centre for Advanced Research in Welding from “Dunarea de Jos” University of Galati, and a portable magnet-arc device, made by two half-diks, that can be manually operated [30]. The pneumatically operated ROTARC welding equipment was designed to reduce the apparatus weight and to improve the efficiency of the forging process applied in the last stage of the Magnetarc welding. The transversal compact magnetising system, employed in the ROTARC welding equipment, comprises several tiny coils: the transverse magnetising field being created by the magnetic support (1), the radially positioned coils (2) and the cores (3), as Figure 9 shows.

Schlebeck E et al. published several results in terms of the magnetically moved arc welding technique with pressure force, design and development of MBL-P or (MIAB) welding, equipment requirements and power sources, as well as welded joints’ quality [31]. Johnson et al. [32] reported the results achieved in MIAB welding of mild steel tubes, having 51 mm diameter and 3 mm wall thickness. In order to assess the quality of MIAB welds, they performed bend tests and measured the internal upset height. Additionally, the authors studied the industrial applicability of the MIAB-welding technique and the main welding factors, such as the upset force, arcing time, and current [33].

The MIAB-welding setup uses permanent magnets that create a stable magnetic field and reduce the electromagnets bulk. At the beginning of the process, electromagnets with an exciting coil with 960 turns, made of Cu, and air as medium are used for generating the magnetic field. A constant current DC power source provides an exciting current to the solenoid. The solenoid coils are magnetised, so that identic magnetic polarity on the edges is obtained. This causes the magnetic flux, that is formed in the two tubes, to be directed against each other [34,35].

The effective design of the MIAB electromagnetic system is a crucial task, since the magnetic flux density affects the arc rotation and the joint quality. The magnetic flux density depends on the exciting current, position of the exciting coil, gap between the workpieces, and the relative permeability of the material. The maximum linear speed of the arc movement is 870 km/h and depends directly on the radial magnetic flux density. The spinning arc, combined with the influence of the metal’s thermal conductivity, develops the uniform heating of tubes’ edges. In the upset phase, the molten metal flashes out, then the plasticised material from the contact area of tubes flows, producing a transversal burr-like barrel shape, and, finally, forming the weld [12,34].

A two-dimensional finite element model was proposed by Kim et al. [34] to study the magnetic flux density distribution determined by the electromagnets in the welded joint. Since a greater magnetic field generates a higher force on the arc that determines higher arc speed and more significant heating, the researchers focused on finding the correlation between the quality of the steel pipes joint and the strength of the radial magnetic field. To measure the flux density at various distances from the outside of pipes and from the exciting coil, a Gauss meter was placed in the middle of the joint. The authors concluded that it is crucial to maintain the highest flux density in the joint area in order to achieve optimal weld quality. Moreover, the design of the electromagnet system and the exciting current delivered to the electromagnets play a significant role in obtaining good joints. It was noticed that the gap size between the pipes and the medium relative permeability have a strong influence on the magnetic flux. They demonstrated that when increasing the permeability and shrinking the gap size, the magnetic flux density increases. Additionally, they suggested that the electromagnet systems may be designed using their analytical approach results. Taking into consideration the heat generated by Joule effect and heat losses by convection and radiation, Glickstein [35] developed a model for investigating the impact of various gases and their mixtures on the joint quality. It is obvious that a full understanding of the phenomena and of fundamental principles of modelling, specific to the welding field, are absolutely necessary to create reliable models, as several of them were discussed in Section 2.4.

A high-quality joint is achieved when maximum flux density is produced in the gap between the faying surfaces [10,20,21,27]. The arc rotation speed depends upon the magnitude of the welding current and of the magnetic field. Drops that may be formed during every cycle have to be avoided and that is why the high speed of the arc rotation is critical in this welding process. Moreover, to increase the temperature of the weld to solidus temperature and to generate a short arc heating phase, the speed must be optimal. Also, the arc rotation speed was also observed to be related to the variation of the arc position, along the wall thickness, as a function of time. The authors reported [23] that the arc rotation speed changed rapidly, simultaneously with the modification of the arc position. It was also observed that in case of thick tubes, the arc rotates with a slower speed around the inner surface, compared to the rotation on the outer edge of the tubes. The variation of the arc speed before the upset stage, caused by the temperature gradient, may affect the joint quality [23]. On the other hand, during the ignition state, the arc is pushed to the inner diameter, due to the development of the magnetic blow effect. This phenomenon is caused by the interaction of the arc magnetic field with the tube geometry, and, thereby, a high-gradient external magnetic field is generated. This strong magnetic field, generated around the outer diameter, blows the arc towards the ID. The study’s results suggest that the arc is blown to the regions that have been firstly heated and the magnetic field has encountered the least magnetic resistance. The continuous heating of the inside edges, until the Curie temperature is reached, makes the gradient of the magnetic field reduce. This phenomenon is attributed to the fact that the iron gradually loses its magnetic property, as the temperature increases, and the arc is then pushed outward by the magnetic arc blow [23,24].

Vendan, S.A. et al. [8,36] designed and developed a MIAB-welding module that was employed to investigate the magnetic flux density distribution and the electromagnetic force, by modifying the MIAB process parameters, in a study case focused on joining T11 steel tubes of OD 47 mm and 2 mm thickness. The experimental module was supplied with a welding current of 400–500 A, and voltage of 90–120 V. A variable DC power source of 75–200 V and 0.1–0.5 A was employed to excite the electromagnetic system realised with a laminated core. Using the welding module, the authors carried out the fusion of surfaces, with a 60% melting of edges and an upset pressure of 30 to 100 MPa. A modified system with a solid core solved the magnetic saturation issue, resulting in a uniform and faster arc rotation. The authors concluded that the optimal parameters for joining the metallic tubes described above are the following: 100–110 V magnetic coil voltage, 500 A welding current and 0.5 A exciting current. It was noticed that a higher exciting current determined an increased rotation speed and an enhanced heating process.

Arungalai Vendan, S. et al. [36] investigated the MIAB-welding process on a prototype module with electromagnets, and addressed the arc behaviour in the case of using permanent magnets. The instability of the system, as well the bulkiness of electromagnets, revealed the need for an auxiliary power supply, the solution being the use of permanent magnets for generating the magnetic field. The authors also designed and developed a mechanical clamping system and a pneumatic control system of the forging process. By reducing the forging phase time, in comparison with the conventional mechanical forging method, this innovative system can be considered an advance in the MIAB-welding process automatization. Based on the comparative parametric evaluation and interdependency study, for both variants, it was confirmed that the welding current, magnetising current and the magnetic field distribution in the air gap are the main parameters which have a crucial role in ensuring the arc stability and the high arc rotation speed.

The operating range related to the experimental process parameters, in joining T11 Cr-alloyed tubes by MIAB welding, was another subject addressed by Vendan, S. A. et al. [22,37]. They used the software package STATA 10.1 to analyse the influences and to determine the optimal parametric window of process parameters as a scatter matrix. Based on the experiments, it was established that the welding current range should be between 200 and 500 A to perform the material heating. Furthermore, the authors noticed the significant role played by the energy loss by convection and radiation heat transfer developed during the process. An appropriate voltage level for setting up and sustaining the arc was experimentally determined to be about 25 V. The voltage induced in the magnetic coil does not have a significant influence in controlling the welding process performance, while the current generates and controls the radial magnetic field.

To optimise the operating process parameters, Norrish, J. et al. [38] simulated the application of the MIAB-welding technique to joining small-diameter thin-walled pipelines, which are employed in gas transmission and distribution. According to the authors, the heating uniformity of surfaces is obtained if the upper limit is restricted up to a 19 mm wall thickness. They concluded that the simulation method can be considered a reliable solution to predict the parametric distribution and the design validation of magnetic path configurations that generate a uniform heating of the workpieces. Additionally, the authors designed an experimental testing system for studying the arc performance and the effects of parameters on the arc behaviour.

Fletcher, L. et al. [2] designed and built a prototype of MIAB welding equipment, suitable for joining X42 pipelines of DN150 × 4.8 mm dimension, that may lead to a 12 to 25% decrease in fabrication cost of natural gas pipelines. The quality of joints exceeded the performance requirements described in the Australian standards specific for pipeline welding. Due to the capacity of high mechanisation, the welding system can ensure the replicability of products and shorter welding cycles in comparison to other solid-state joining methods. The authors recommend the standard AS2885.2-2002 for testing the welded joints, and put the emphasis more on the online monitoring, than on the conventional NDT [39].

When tubes with a thickness larger than 6 mm were welded, an unstable movement of the arc in the regions with low magnetic field and non-uniform heating has been noticed. The control methods recommended to address and fix these issues entail the following techniques [1,11]:

1. Mechanical solution: one tube is fixed and the other, coupled with an engine, rotates around the other with an amplitude similar to the wall thickness. The orbiting frequency was suggested to be maintained at nearly 1/100th of the arc rotation frequency, so that, during the heating phase, the arc is distributed across the entire wall thickness of both tube faces. Following the heating, the two surfaces are rapidly aligned to be efficiently forged, and, further, welded together.
2. Electromagnetic solution: positioning the conductor inside the tubes to be welded. The interaction of the magnetic fields determines the arc movement across the tube ends, and then the uniform heating.
3. Significant axial magnetic field: the magnetic field can be adjusted to obtain a stronger axial component than the radial one. The axial component of the magnetic field, in interaction with the radial current component, produces a greater force on the arc, pushing it towards the tube OD, and heating the faying surfaces. After the required heating is achieved, the current and the pressure force are changed, such that the arc scans through the faying surface and moves to the inner diameter prior to the upset phase [9].

Among all measures presented above, due to the precise control over the process and need of shorter welding time, the mechanical solution could be considered more efficient. Furthermore, in this case, the short-circuiting across the tubes is minimised.

**Figure 10:**
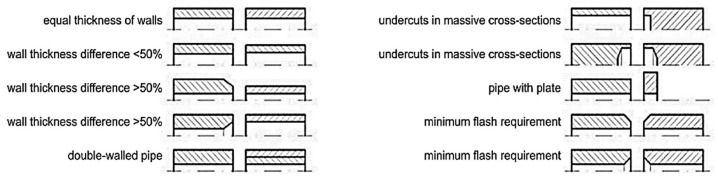
Preparation of materials’ edges [40].

Piwowarczyk, T [40] investigated the effect of pipe faces on the quality of MIAB-welded joints. The experiments focused on modifying the power transmission elements, as well as on the metallurgical and mechanical characterisation of joints. In order to prevent the formation of imperfections in welds, the surface of thin-walled elements must be correctly prepared by chemical cleaning. Several preparation variants of materials’ edges, typical for the automotive industry, are shown in Figure 10.

**Figure 11:**
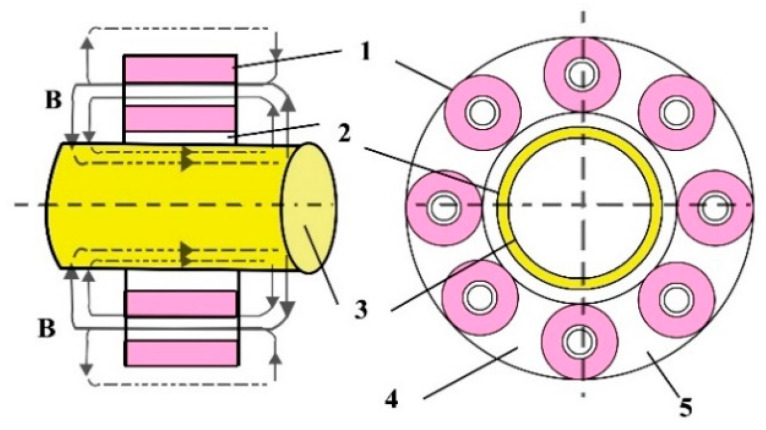
Original longitudinal magnetisation system with multiple solenoids. Reproduced from [10], Journal of Materials Processing Technology, with permission from Elsevier, 2010.

The deformations developed during welding must be analysed, because some of them may be suitable for certain industrial applications. Moreover, as an effect of the upsetting, it the formation of a fish mouth in the central part of the weld was reported, but no cracks were observed. Additionally, a bevel weld and weld necks may cause a variety of flash forms and spatters, meaning imperfections in the weld. However, these imperfections have a negligible effect on the microscopic properties and on the hardness measurements. Iordachescu, D. et al. [10] generated the optimum parametric window for the MIAB welding of ST37 with 25.4 mm OD and 3 mm thickness, using a modified magnetisation system with multiple solenoids that were mounted in two independent shells, parallel with the longitudinal axis of the tube. Eight small peripheral solenoids were connected in series, with four coils positioned on each half shell (Figure 11).

Using two half shells, the positioning and removal of the magnetisation system, even for longer tubes, are much better facilitated. This modified longitudinal magnetisation comprises a peripheral solenoids system (1), gap (2), thin walled tube (3), half-shells (4,5) and promotes a concentrated magnetic flux (B) distribution with a positive effect on the arc rotation stability and portability. Based on the experimental study and on the analysis of the upsetting force, welding current, arc rotation time and resultant magnetic field, the authors assessed the performance and the efficacy of the MIAB-welding process to be applied for achieving quality joints [10].

**Figure 12:**
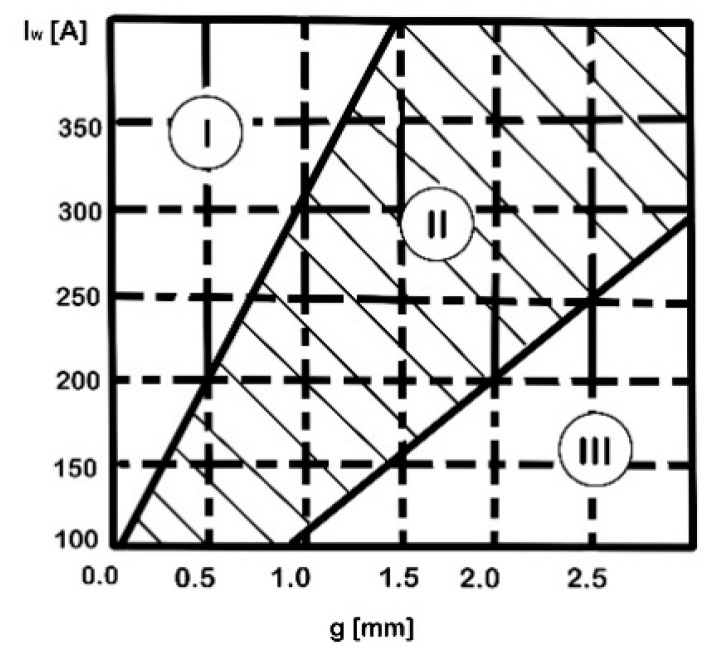
Arc stability zones vs. different gap lengths and welding current. Reproduced from [10], Journal of Materials Processing Technology, with permission from Elsevier, 2010.

A deformation factor, computed as the variation ratio of the total standoff (after upsetting) and the initial total standoff, was defined as a relevant indicator of the weld quality. Applying the optimum upsetting pressure and arc rotation time, a deformation factor of 0.5 indicated a good quality joint. Using a high-speed camera and an optical sensor, the influence of the tube gap on the arc stability and the effect of the magnetic induction on the arc rotation and heating duration was analysed, as well as the modification of the arc rotation velocity versus the welding time. Figure 12 exhibits the arc stability diagram, where region II is considered the stable arc region, with the optimum gap length correlated with the welding current range. The instability of the arc, specific to the regions I and III, is caused by a too small or too big gap length, respectively.

Ku-Jin Mo et al. [41] proposed a coil system that can be opened to generate the magnetic flux and a 3D numerical model for analysing the development of the electromagnetic field. It was observed that the resultant magnetic flux distribution was dependent on the exciting current in the coil and the position of the coil from the outer surface of the joint. Moreover, it was found that the arc rotation was strongly influenced by the tubes gap and welding current.

Additionally, for the common applications of the MIAB-welding process, the researchers investigated some unconventional cases, such as the joining of non-ferrous metals, pipes with a non-circular configuration, or hollow and solid parts. For instance, Mori S. [4] proposed a solution for the MIAB welding of non-ferrous materials, such as Al to Al and Al to Cu, using shielding gas which has an important role in preventing the oxidation phenomenon of the Al surface [42,43]. The technical solution consists of using ferrous pipes reinforced into the Al pipe core, with the aim to generate the effective magnetic field. Employing a constant voltage DC power supply, the melting process of the pipes’ ends depends on the current polarity. On the positive side of the Al-Al joint, a contamination of the butt surface by oxides adhering to the surface was noticed, while on the negative side, a cleaner and a consistent melted volume was observed. In case of joining Al to Cu, a technical solution could be the placing of the Al pipe on the negative polarity side, avoiding, in this way, the oxidation of the Al pipe surfaces. Another solution is the use of mixed shielding gases, such as H_2_ with Ar, resulting a higher arc temperature and a better heating of surfaces, due to the presence of H_2_.

MIAB welding can also be applied for the joining of non-circular parts like squares, rectangular octagonal shapes, or D sections [1]. To avoid the uneven heating of the sharp corners or the lateral movement of straight sides during forging, a magnetic system positioning and an efficient clamping mechanism may be required. An example of such a component that can be joined by MIAB welding is the rear axle of an automotive that has round and square sections at each end. Lines of an Beel agricultural elevator require welding of 44 × 15 mm oval tubes to plates in 1 s time. Other applications of MIAB-welding tubes to plates are the mounting plates for wall brackets, end fitting of racking systems and bell cranks. The issue, in terms of the difference between the thermophysical characteristics of the tube and plate materials, can be solved by using high-amperage and short-arc heating cycles.

Taking into account the intercorrelation between the cross-section and arc spot diameter, Kachinskiy V.S. et al. [11] proposed a control method for an efficient heating of thick-walled tubes and solid parts. The welding of solid rods, with diameters up to 25 mm, was demonstrated to be feasible. An intensive metal evaporation that acts as a shield in the gap space and prevents the oxidation of melted parts was also reported [11].

Based on the numerous studies, the scientific literature provides comprehensive guidelines for designing MIAB laboratory welding module and for optimising the process parameters in order to achieve high-strength and -quality welded joints for specific industrial applications, with certain materials and dimensions. In conclusion, an efficient and reliable MIAB-welding process needs an appropriate design of welding modules for the making of good welds. One of the main objectives of the experimental and simulation studies, developed and published in the scientific literature, was to identify the optimum parameters that can ensure an adequate joining process. The design calculation methods, which explain how the metal properties influence the electrical power, revealed inadequate requirements for the magnetic field and mechanical input. Furthermore, the heat sources were considered fixed in the simulation studies and that may fail to explain the changing of the thermal gradients and the actual dynamics involved in the process. Further research on design equations, which should consider the moving heat source and heat lost in the mathematical model, will certainly improve knowledge in the welding field.

## 4. Experimental, Parametric and Numerical Analysis

Studies for monitoring the process parameters have been developed by using high-speed cameras [19], Gauss meters [12,14], thermocouples [14], and voltage probe techniques [14,21]. Based on the finite element modelling method, several simulation studies, addressing the prediction of thermal and magnetic field variations during welding, have been performed by several researchers [8,13,37]. The main aim of the studies was to establish the process concepts and, further, to make the optimum decision in terms of parametric range for the efficient welding of various materials and geometries. The input data in designing the experimental procedure comprise the following process parameters: welding current, upset current, heating time, upset time, and magnetic coil voltage. The output variables are either the magnetic flux density, process temperature, arc speed, or directly the weld features like strength, geometry, microstructure, and residual stress developed in the welded joint. Substantial thermal expansions and contractions, as well as the metallurgical phase changes, caused by the heating and cooling cycles developed during the welding process, and, further, modification of material mechanical characteristics determine the occurrence of significant residual stresses in the welded joints. Consequently, factors, such as thermal, metallurgical and mechanical properties, play a crucial role in the ultimate distribution of residual stresses in the welded joints. Several results of the parametric analysis for various experiments are presented and described in Table 1.

Briefly, the experimental and simulation works have addressed the parametric interaction effects on weldments. On the other hand, the constraints and technical challenges for the dynamic process conditions, involving the rotating arc and heating effects, are insufficiently studied. The continuous change in the arc position and length influence on the heat input, as well as the air dynamic resistance, developed by the rotating arc, are not investigated in detail. To achieve quality welded joints, these variations need to be taken into consideration in the optimisation of process parameters. Details on the integration of the “Internet of Things” (IoT) and data acquisition are not available for the MIAB-welding process, restricting the precise control on the arc dynamic. If the aspects mentioned above will be more and better addressed in the future research, then the MIAB-welding technique might be considered a more competitive method for joining ferrous and non-ferrous tubes and, further, the applicability and the demand in the manufacturing industry could increase.

## 5. Characterisation of MIAB-Welded Joints

Because testing standards for evaluation of MIAB welded joints quality have not been developed yet, then standards specific for other solid-state welding processes are used to assess the welds quality. The radiography, metallographic, and X-ray diffraction tests are employed to investigate the macro and micro structure, and the strain level developed during welding. Several results in terms of testing and characterisation of welded joints, performed by MIAB welding, are presented and discussed in Table 2.

The researchers have also analysed the MIAB-welded T11 tubes by Notch Tensile Strength (NTS) and they obtained distinct values for each of the TMAZs, due to their different microstructures. From all tested TMAZs, a single sample that exhibited a granular bainite had the NTS lower than the NTS base metal [57].

Kachynskyi et al. [21] applied the MIAB welding for joining small pipes of up to 212 mm OD and 3 mm thickness which are found mostly in the industry of fittings and pipeline constructions. The results of the mechanical and metallographic testing indicated weld properties almost similar to the base metal properties. MIAB welding increases productivity and allows for the automation of the manufacturing process automation too.

Due to the heating–cooling processes, the temperature continuously modifies and, consequently, the material suffers several phase transformations. The majority of the research articles reported results related to the ferrous materials joining. However, being a solid-state process, the MIAB welding may be applied for joining non-ferrous or even dissimilar materials. The future investigations may demonstrate the feasibility of the process and may conclude that this technique could be seen as a technological revolution, since many other conventional welding techniques have failed in joining dissimilar materials.

## 6. Industrial Applications

The main advantages of the MIAB process are the increased productivity, due to the potential high automation degree, and the lower fabrication cost of welded tubes. The MIAB-welding process can be completely automated after the placement of parts in the MIAB-welding system. Depending on the dimensions and material properties of the tubes, the welding time and current can be set and controlled using a system based on Programmable Logic Controllers (PLCs). The magnetic field and the upset force are controlled by selecting the permanent magnet that depends on the magnetic flux range and the pressure developed by the hydraulic and pneumatic system. This process is suitable for industrial mass production, due to the short time cycle and replicability of quality joints. The MIAB method can be applied to perform butt welding of thin-walled tubes, butt and T-butt welding of automobile parts, thick-walled tubes with some challenges, solid parts, tube-to-plate welding, or tube-to-flange welding.

### 6.1. Automotive Industry

This joining method is predominantly used in the European automotive drive shaft industry for parts of the rear axle assembly, wheel bearing housing, pipe and tube assemblies, shock absorber assemblies, threaded sleeves assemblies, nuts welded to plates, and brake pipes [37,38].

The efficiency of the MIAB-welding process in joining automotive components of dissimilar materials, like stabiliser bars, was studied by Peng, M. et al. [58]. For instance, Ductile Cast Iron (DCI) tubes and E355N alloy steel were welded by this technique without preheating or need of filler material. Applying traditional welding techniques, peeling and joint deformation, caused by the poor weldability of the DCI material, can occur in the welded joint.

Kumar, S.R et al. [5] presented the results of joining T11 to T91 boiler-graded tube materials with 3.5–5.5 mm thickness and 44.5 mm OD, typical for engine applications. Apart from the mechanical properties, high corrosion resistance and embrittlement resistance to reduce the expense of damage and replacement of parts are required. These conditions are met by the ferritic martensitic steels, such as the combination of T11 and T91 materials. For the joining of T91 steels, the temperature must be maintained at an optometric range to prevent brittle and hard microstructure and crack openings.

Kuchuk-Yatsenko, S.I. et al. [54] reported significant information related to the applicability of MIAB technique in automotive industry, particularly for the shock absorber, steering rod, and torque rod parts, respectively, using the MD-101 and MD-1 welding systems, as well as the parameters presented in Table 3. To achieve steering rods, pipes of 300 mm in length with hollow rods of 60 mm long thread need to be welded on both sides. To fabricate shock absorbers, welding is performed on 300 mm length pipes, with 0.02 mm inner Cr coating, and 60 mm long thread on one side. The tensile and bend testing of the MIAB-welded parts showed high mechanical strength, fatigue resistance, and good ductility, without other supplementary mechanical operations. According to the procedures adopted in the automotive industry, Destructive Testing (DT), such as full-scale tensile testing and local bending of circumferential welds, should be performed.

The study made by Balta B. et al. [59] describes the design and testing mechanisms for truck chassis brackets carried out by the MIAB-welding procedure. Based on the parts’ natural frequency and road load test data, the welded parts’ design was analysed using the FEA technique and was used to establish the optimum correlation of process parameters. The tightness of the welded parts achieved using the MIAB-welding technique proves the suitability of the process to weld pressurised vessel parts. FEM was used to develop the numerical model for a fender assemble and for estimating the stress distribution. It was found that the clamping brackets are under higher stress predicted in the fender brackets. The authors reported a good agreement between the numerical and experimental results.

In [60], Balta B. made a comparative study of the MIAB welding with Continuous Drive Friction Welding [CDFW] employed for joining medium carbon steel tubes and forging brackets. This kind of joint is specific to the drivetrain applications from the automotive industry. The MIAB-welding method has been found as a cost-effective alternative to the CDFW procedure.

D.A. Edson studied the application of the MIAB-welding method, frequently used to join components in the rear axle casing of Ford Transit car, by performing two circular or square-butt joints [61].

Hone P. N. [62] reported multiple and potential applications of this joining procedure in the automotive industry. For example, non-circular tubes occasionally needed specialised magnets with particular shapes, propeller shafts, automotive axles, gasoline tank vent pipes, drive shafts, shock absorbers, and gas-filled struts were only several industrial applications. The axle tube and the centre housing of the Ford Transit van axle, as well as the Vauxhall Astramax’s rear axle were joined by this method too.

Hagan et al. [63] described the use of MIAB welding for the production of the Fiesta rear axle cross-tube assembly in the Ford Motor Company. On the list of potential welding techniques to be applied, more popular welding methods, such as friction welding, flash welding, and GMAW, were taken into consideration, but issues such as an inability to maintain the radial connection between the shaped flange spindles and the axle tube, the alignment between components or the strain and stress field developed in fusion welding, were the main arguments for choosing the MIAB welding in detrimental of the procedure mentioned above. After optimising the process parameters, performing all essential tests and implementing the quality monitoring procedures, the MIAB technology was successfully implemented in mass production.

Hiller et al. [64] presented the application of MIAB welding in the fabrication of truck cab suspension components. A cast iron lever was welded to an extruded steel torsion tube and the authors noticed the benefits of this process, such as short welding time and great mechanical properties of the solid-state joint developed between cast iron and steel.

Thomas Bschorr [65] described the application of MIAB welding for the composite structures of steel and nodular graphite cast iron (S235/EN-GJS-400-15, S355/EN-GJS-600-3). They highlighted the specific issues found when the conventional fusion welding process was employed to achieve the composite structure from the vehicle construction or from the fittings industry. The composite structures achieved by MIAB welding are characterised by high dimensional accuracy, good repeatability and resistance to dynamic loads.

### 6.2. Tubular Elements Used for Pressure Components

Faes, K. et al. [66] developed the MIAB-welding technology, and a control mechanism for the cost-effective welding of High-Strength Steel (HSS) tubular parts of a hydraulic cylinder that works under the pressure of 250 bar. This type of cylinder has a bottom with different structural forms and chemical compositions, an OD up to 200 mm and a wall thickness up to 10 mm. The control mechanism was designed to operate in the plastic deformation stage, wherein a short-term increased current is essential for a further increase in temperature on the surfaces to be welded.

Vendan, S.A. et al. [67] performed experimental tests for checking the feasibility of the MIAB welding, applied to join carbon steel tubes of 44 mm and 51 mm OD and 5–6 mm thickness that are used for fabrication of economiser coils from the boilers and pressure vessels. The mechanical testing results and the metallurgical characterisation of the welded joints confirmed the good weld strength and the integrity of high-pressure parts, in accordance with the AWS B.4 standard [68].

Isravel R.S. et al. [69] tested the MIAB weldability of SA210GrA tubes which are used in boiler applications. They concluded that the defects observed on the first samples were caused by the insufficient maintaining of eccentricity and so the generated current could not produce a proper fusion. The authors reported that minimum current of 450 A is needed to obtain quality joint between SA210GrA tubes.

### 6.3. Oil and Gas Pipelines

Fletcher, L. et al. [2] demonstrated the advantages of employing the MIAB-welding process for highly automated factory production lines, wherein pipes with diameters from (DN700) 75 mm to (DN450) 450 mm and thicknesses up to 10 mm can be joined. These pipelines can be welded within 10–15 s, meaning 7.5 km of welding per day. A significant advantage is the fabrication cost which is reduced by approximately 15%. Taking account of the process productivity and reduced cost of products, MIAB has been proposed to be implemented for the high-integrity joining of oil and gas transmission pipelines.

Tagaki et al. [70] developed a rotating arc welding device for the fabrication of gas pipelines. The equipment was employed to weld mild steel gas pipelines with an outer diameter of 60.5 mm and 89.1 mm, and wall thickness of 3.8 mm and 4.2 mm, respectively. The researchers demonstrated that high-quality and reliable welds can be produced with high efficiency.

Other researchers, such as Norrish, J. et al., stated that MIAB welding is an efficient girth-welding process and reported that this technique can be extended for welding pipes with wall thicknesses of 19 mm [37].

According to Schelbeck E. [31], the magnetically moved arc with pressure (MBL-P) or MIAB welding can be applied to fabricate CO_2_ pressure cylinders for fire extinguishers, pipe screw joints for hydraulic lines, and pipe–flange joints of 32 to 85 mm nominal width. The use of MIAB welding for unique applications, such as the drive component for a car, or for a chain-type carrier used in beet harvesting equipment, and T-butt joints of hollow sections, pipe–plate, and section–plate, is also mentioned by the authors. It was pointed out that the MBL-welded joints, and welded pipe–flange couplings are characterised by high strength, proved by the results of fatigue strength testing.

According to Andreas Jenicek et al. [71], a procedure with specific economic viability, such as an enhanced version of MIAB welding, might be used to join tubular hollow bodies, like nuts, sleeves, and bushes, to sheets. Internally threaded Al components (M8 and M24) were welded onto perforated sheets of ENAW-AlMg3 and ENAW-AlMgSi1, using Drawn Arc Stud Welding (DASW) devices. The applicability of the MIAB-welding method to join Al to Al and Al to Cu connections was studied by Shuzo Mori et al. [4]. In this case, it was harder to join non-ferrous materials than ferrous materials, because of the challenge to reach the necessary flux density. However, the pipe is usually filled inside with iron core in order to achieve quality non-ferrous welded joints.

Hassel Thomas et al. [72] investigated the feasibility of the MIAB-welding technique for joining the L80 casing material and corrosion-resistant duplex steel to fabricate geothermal borehole liners and, finally, they achieved dissimilar joints with good mechanical and corrosion-resistant properties. The metallurgical challenges were overcome by Ramesh, S et al. [56] who analysed, using SEM and radiography testing, the structure modification in the welded samples of SAE 213 T11 and T91 dissimilar boiler-graded steel tubes. The dimensions of tubes were 44.5 mm OD and 4 mm thickness.

The MIAB-welding method has been predominantly adopted in the European automotive industry for joining ferrous tubes that are employed in the manufacturing of various parts. It has also been explored in the power industry for the manufacturing of heat exchangers, boiler tubes, and other high-pressure parts which find utility in the corrosive and hazardous environment. Available reports for the MIAB welding of dissimilar steel grades like T11–T91 or non-ferrous materials, like Ti, do not provide sufficient information. The process feasibility studies for the non-ferrous materials can lead to increasing the acceptability level and to widening the applicability of this joining process to the aerospace industry, where the non-ferrous materials’ durability and light weight are essential requirements. The present state of the art focused on the MIAB-welding process and its applicability in several industrial sectors provides a vision in terms of extending this interesting welding to other industrial applications. In conclusion, researchers should further explore the suitability of MIAB welding for joining different non-ferrous materials and dissimilar materials.

## 7. Conclusions

This review article covers the evolution of MIAB-welding systems, various designs and operational aspects, limitations and advancements, welded joint testing and characterizations that are typically required as per industrial standards. Based on this critical state of the art, MIAB welding is evident to have the following advantages over the conventional solid-state welding techniques:

- Short welding cycle.
- No rotation of parts to be welded, so the alignment can be maintained.
- Uniform heating of the joint results in low distortion.
- Welding a wide variety of ferrous and non-ferrous materials.
- Minimum loss of material.
- Low residual stresses.
- No shielding gas requirement.
- Low energy requirement and, consequently, cost-effectiveness.
- No formation of intermetallic in the fusion zone.
- No melting and, therefore, no significant change in material properties.
- High potential to automate the process and, subsequently, high reproducibility of welds.
- Similar strength and ductility properties of welds and base metal.
- No pores, inclusions or other volume defects frequently found in fusion-welded joints.
- Insignificant dimension (no more than half of the pipe wall thickness) of the outer and inner reinforcement, which is uniformly distributed around the perimeter being welded.
- Easy automation and no requirement for a highly skilled operator.

A thorough analysis of the MIAB-welding literature yields some recommendations on analytical, modelling design/developmental, and applicational aspects. The investigations covered the mechanics, design philosophies, methodology, and techniques for the MIAB-welding equipment. Design methodologies for the MIAB-welding process are not sufficiently covered in the published research reports. Based on the present extensive study, the following observations were briefly synthetised:

- The preliminary MIAB-welding process setup has undergone significant transformations in terms of the magnetic field arrangement, forging method, and clamping configurations by taking account the material geometry.
- It has been found that the welding current, tube gap, magnetic field density, and arc rotation time are the key governing process parameters that have a strong influence on the metallurgical behaviour on the weld strength.
- Arc current is responsible for required heating of the weld surfaces up to the solidus temperature to achieve the plasticizing of the material, while the upset current affects the expulsion in the upset stage.
- Arc upset current controls the width of the TMAZ in the process as it governs the expulsion of the excess material and the surface impurities. Increased upset current results in reduced width of the TMAZ region.
- It is a challenge to join tubes with a thickness larger than 6 mm. However, several technical solutions to address the issues occurred in this type of applications have been presented in this work.
- The replicability remains a challenge in case of several industrial applications due to the variation of process parameters and welding conditions.
- Oil and gas pipes with diameters up to DN450 and thicknesses up to 10 mm can be welded using the MIAB technique. High-integrity joints can be fabricated using the MIAB-welding method at extremely high production rates.
- Small wall thickness and low-pressure applications represent the majority of the MIAB-welding applications. According to the studies, the non-ferromagnetic materials, like Al and Cu, were welded using the MIAB method that is currently applied for joining the ferromagnetic materials.
- This technique can be employed for welding several alloy steel grades like T11, T91, mild steel or a combination of these alloys. It is also being explored for welding similar or dissimilar non-ferrous materials such as Al to Al or Al to Cu.
- MIAB welding is an appropriate method for welding, in 10–15 s time, pipes with an outer diameter from 75 mm up to 450 mm and a 10–35 mm wall thickness.
- By implementing this joining procedure, direct labour expenses and labour-related overheads, like training costs, can be significantly reduced.
- Process limitation comes from the arc (of finite width) that is initially rotating on the inner edge and then predominantly on the outer edge of the tube faces. As a predictable result, uneven heating and consequent poor-quality welds are obtained. This radial movement of the arc path occurs no matter the wall thickness, but becomes less consistent when the wall thickness increases above 6 mm.
- An issue encountered in welding thick wall tubes is in ensuring the immediate arc rotation to avoid local melting and the arc short-circuit.

The research work for establishing an optimal parametric window to achieve good welds, even with marginal offsets, needs to be extended. Progress in this direction will help establish the dependencies of the process parameters, conditions, material properties, and the weld quality. This is essential to project this joining technique as the preferred method in various industrial manufacturing applications. This review paper highlights the research gaps to be filled and the possible investigation directions to identify solutions that would make this process adaptable and appropriate for the manufacturing industry, as follows:

- Three-dimensional FE numerical models to calculate the heat distribution, electromagnetic force distribution, or magnetic flux distribution have not been reported so far.
- No research on the electrical circuit modelling of the MIAB-welding method has been carried out so far.
- There is not yet any available information on the development of a laboratory prototype MIAB-welding module with a compact power supply for comprehending and investigating the impact of different input parameters on the arc rotation.
- The testing standards and codes, specific to testing the MIAB-welded joints, are not yet established for most of the industrial applications.

An extension of this joining procedure for higher acceptability in industry needs extensive research work for establishing an optimal parametric window, useful to achieve good welds even with marginal offsets. This research work highlights the research gaps to be filled and the possible directions to attain solutions that would support a larger implementation and adaptability of the process in the manufacturing industry. The justification of this review is based on the promising features of the MIAB-welding process as it was demonstrated and reported for the alloy steel and carburised tubes.

## Figures and Tables

**Figure 13:**
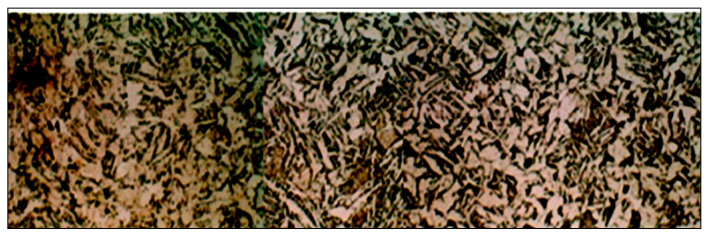
Weld (right side). Reproduced from [10], Journal of Materials Processing Technology, with permission from Elsevier, 2010.

**Figure 14:**
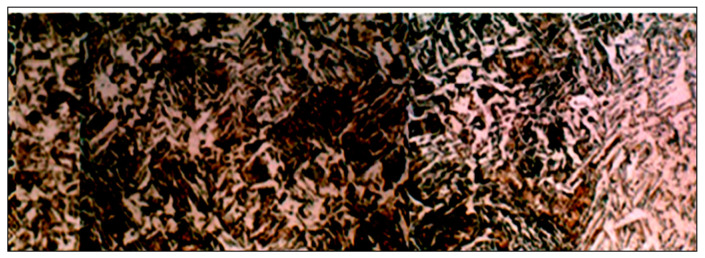
Weld (left side). Reproduced from [10], Journal of Materials Processing Technology, with permission from Elsevier, 2010.

**Figure 15:**
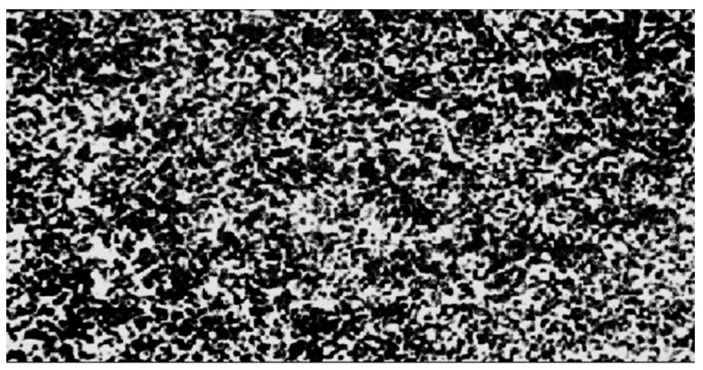
HAZ with partially and completely recrystallised region. Reproduced from [11], Welding in the World, with permission from Springer Nature, 2002.

**Figure 16:**
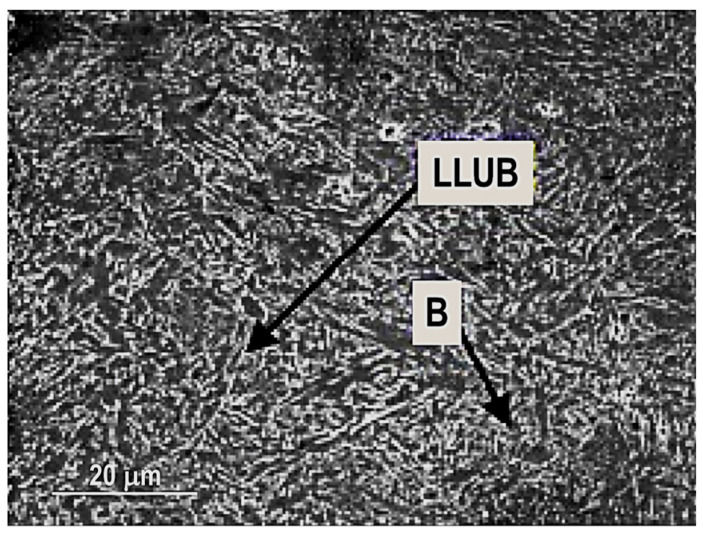
SEM image of TMAZ1. Reproduced from [5], Materials Today: Proceedings, with permission from Elsevier, 2020.

**Figure 17:**
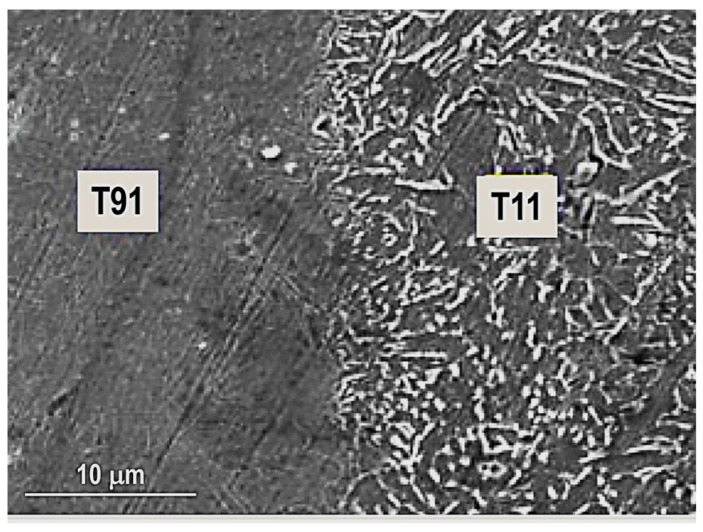
SEM image of WI. Reproduced from [5], Materials Today: Proceedings, with permission from Elsevier, 2020.

**Figure 18:**
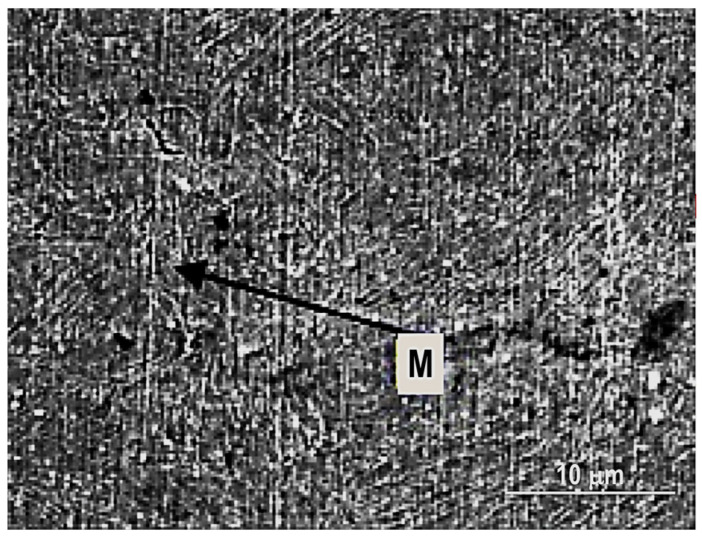
SEM image of TMAZ3. Reproduced from [5], Materials Today: Proceedings, with permission from Elsevier, 2020.

**Figure 19:**
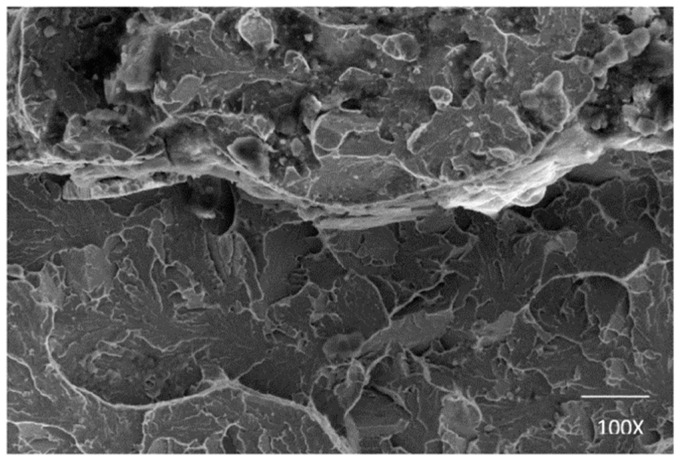
SEM Microstructure [45].

**Figure 20:**
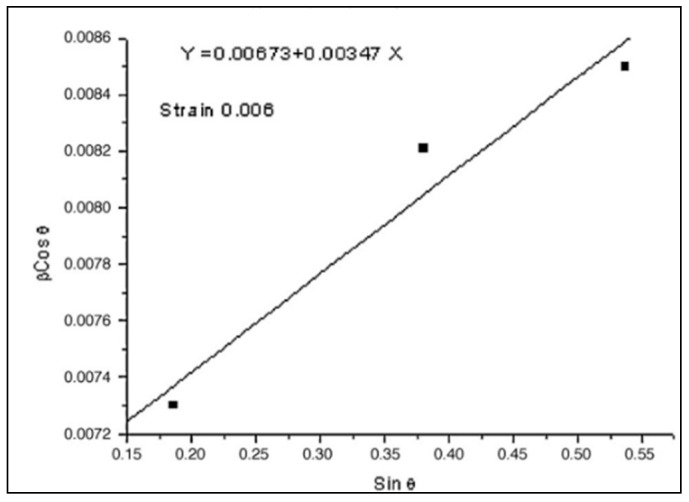
W-H Plot of weld region [49].

**Figure 21:**
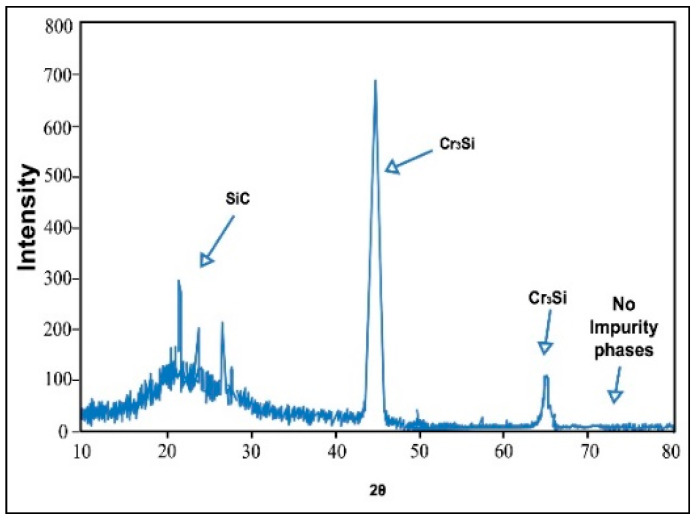
XRD Spectral analysis of weld region [49].

**Figure 22:**
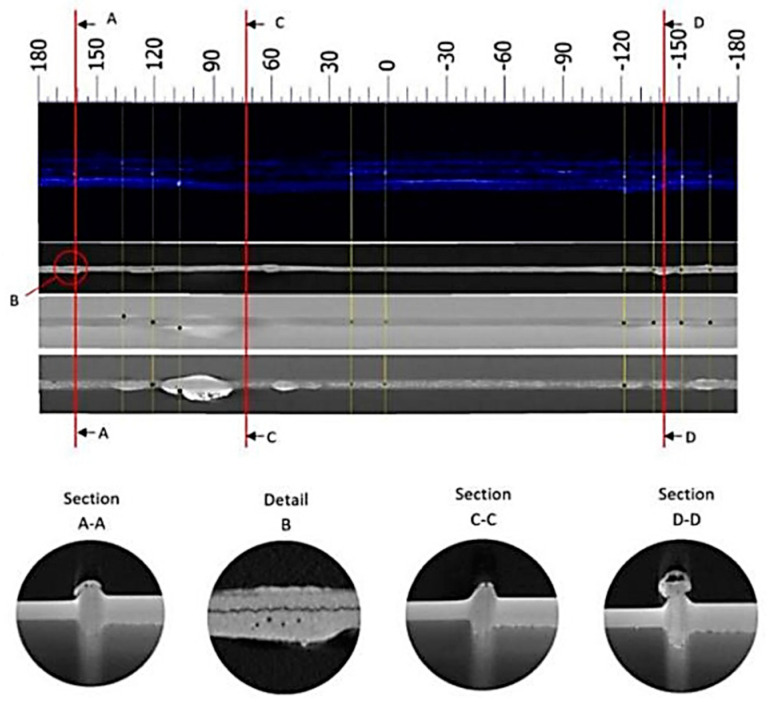
Comparison of B-scan and CT results in the cross sections of the shaft A–A, C–C, D–D and detail B [55].

**Table 1:** Results of parametric analysis for various experiments.

Application	Parameters Considered in the Analysis		Simulation/ Optimization Tool/Test Method	Observations	Remarks
	Input	Output			
A213(T11) tubes [6]	welding current, welding voltage, magnetising current, magnetising voltage	arc speed, weld quality	MGGP framework implemented on GPTIPS	Linear variation of arc speed with arc voltage and welding current, parabolic variation with coil magnetising current.	Welding current is the most significant factor.
T91 Steel tubes 44.5 mm OD, 5.7 mm thickness [7]	upset current, welding time, tube thickness	tensile strength, microstructure	DT/NDT	Low thermal effect, high-speed arc rotation variation in HAZ.	Formation of voids caused by Cr percentage in steel. Low UTS at interface between weld and base material.
STK41: JIS G 3444 AA1050 JIS H4080 Cu Alloy C1220: JIS H 3300 60 mm OD, 3 mm thickness tubes [23]	exciting current, arc current, tube gap	arc rotation velocity, arc position, temperature	Maxwell for electromagnetic analysis	Arc rotation velocity varies with thickness and welding time. Temperature modifies with welding current and magnetic field.	High temperature reduces the yield strength of the material. The arc speed and its position are dependent on thickness.
ST37 tubes 25 mm OD, 3 mm thickness [10]	tube gap, magnetic field, arc current, welding time, upset force, upset rate	arc stability, tube deformation, microstructure, hardness, arc velocity	NDT, high-speed photography, optical sensor velocity measurement	Tube gap affects the arc stability; the upsetting force and its rate (dynamic effect) assure the weld quality.	Larger magnetic field with a new peripheral magnetic system. The molten metal flashes out, indicating some fusion weld characteristics. Arc velocity increases from zero, undergoes fluctuations, and then becomes quenched.
SA335, grade 11 pipes [26]	upset current, volumetric dilation, plasticity	residual stress, microstructure	ABAQUS, ANSYS	Welding current value modified: high for ignition, low for uniform heating, and high in the upset stage. Plasticity reduces the dilation effects. Welding time increases the residual stresses. Pressure decreases the axial stresses.	Phase transformations determine the circumferential residual stress.
Mild steel pipes 48.1 mm OD, 2.0 mm thickness [34]	exciting current, gap centre to coil distance, gap length	magnetic flux density	FEM—ANSYS 2D	Magnetic field depends on the exciting current and is inversely proportional to gap length and gap centre to coil distance.	Flux density drops inside the pipe rapidly, and the relative permeability has a small effect on the magnetic flux density distribution.
Carbon steel (SA210) tubes 44 mm OD, 4.5 mm thickness [44]	upset current	tensile strength	NDT	Formation of decarburised layer, when the upset current is low, may cause weld fracture. A high upset-current value determines the high-strength region.	Minimum upset current requirement is 800 A.
A213(T11) steel tubes 54 mm OD, 7 mm thickness [45]	welding current, welding time upset current	weld geometry, penetration depth, tensile strength	PI-PID Controller studied with Ziegler-Nichols tuning	Weld bead width is uniform. Depth of Penetration (DOP) UTS increases with the upset current.	The designed double controller reaches the set point faster than the conventional controller. Mechanical, electrical and control analysis determine good stability and reliability.
SA335, grade 11 tubes, 33 mm OD, 3 mm thickness [46]	welding time, upset pressure	mechanical and structural properties, residual stress	ANSYS	Positive influence on mechanical and structural properties. Negative influence on residual stress field.	Residual stresses cause solid phase transformations that reduce longitudinal stresses and volumetric phase change.
A213(T11) tubes, 47.6 mm OD, 6.6 mm thickness [47]	welding current, gap length, magnetic field	weld bead and penetration, arc behaviour	DT/NDT	Welding current, upset current and flux density determine the arc behaviour and weld features.	Optimal value of arc current = 200 A, upset current = 1100 A, upset time = 0.3 s.
A213(T11) tubes, 48 mm OD, 6 mm thickness [48]	welding current, welding time, upset current	tensile strength, ductility, microstructure	DT/NDT	Void formation caused by the low welding current, high upset current, and welding time. Welding current is the most significant factor for the weld quality.	Interaction effects of welding current and upset current significantly affect the microstructure and weld tensile strength.
A213(T11) tubes, 47.6 mm OD, 6.6 mm thickness [49]	welding current, welding time, upset current	DOP, reinforcement, weld bead, weld geometry	DT	The combined effect of welding current and welding time on DOP and weld bead. Low welding current, high welding time; and high arc current, low welding time are the combinations for the adequate heat input.	A minimum gap between tubes is required to avoid short circuits and arc extinction.
ST37 low carbon steel tubes, 25.4 mm OD, 3 mm thickness [50]	gap length, distance between magnetisation shells, magnetisation current, deformation factor	magnetic field, welding process stability	IR imaging for temp study, NDT, DT	Stability depends on the welding current and gap value. Magnetic field (B) increases with the magnetisation current (Im). Maximum value B occurs at a gap of 0.5 mm, and B decreases for gaps >1.5 mm.	Peripheral solenoids magnetisation system, deformation factor >0.5 required for a good quality joint.
AISI 409 Ferritic SS Tubes, 4 mm Thickness [51]	arc time	weld zone dimension, tensile strength, hardness	microscopic analysis, tensile test	Larger weld zone and increased strength. Refined grain size in the weld region. Increased hardness in the weld region	Increased heat input with the arc duration causes forged structure in the weld zone with increased grain size and axial shortening. Upset stage causes formation of large strain field.
AISI 409 Ferritic SS Tubes, 4 mm Thickness [52]	arc current	weld zone dimension, tensile strength, hardness	microscopic analysis, tensile test	Increased current value resulting in larger grain size, followed by upset to determine grain refinement. No coarsening of grains was observed. No phase transformation in TMAZ.	Increased current enlarged the joint region and axial shortening. Optimum range of current required for proper upset. High-density dislocations cause joint strengthening.
AISI 409 Ferritic SS Tubes, 4 mm Thickness [53]	arc current, upset current, arc rotation time	weld zone dimension, tensile strength, hardness	macroscopic, microscopic analysis, tensile test	Fine-grained and deformed structure in the weld. TMAZ has significant strength in comparison with the base metal.	Parameter variation at 5 levels as per Central Composite Design of experiments. Oxide particles, improper forging and voids adversely affect the weld strength.

DT—Destructive Testing; NDT—Non-Destructive Testing; FEM—Finite Element Modelling; IR—Infra Red.

**Table 2:** Characterisation reports developed for various materials and geometries.

Material, Geometry Dimensions	Process Parameters	Characterisation
A213 (T11) tubes 47.6 mm OD, 6.6 mm thickness [3]	Arc initiating current: 280–300 A. Initiating time: 0.5–1 s. Heating stage current: 200–220 A. Heating time: 20–30 s. Upset current: 1100 A. Upset time: 0.3 s.	Higher UTS of the weld than of the base metal. SEM indicates brittle fracture caused by inadequate heat input or short heating time. Bend tests showed some failed samples because of insufficient pressure, presence of impurities or excess melting. Highest WZ hardness because of high dislocation density and high temperature close to the WI. Impact toughness decreases with increasing heat input.
A213-T91 steel tubes, 44.5 mm OD, 5.7 mm thickness [7]	Welding current: 900 A. Upset current: 1100 A. Welding time: 12 s.	High hardness. Low UTS at high upset current. Computed Tomography (CT) and Radiography Test (RT) results show several defects with incomplete fusion. Lower arc current causes cracks formation at weld interface (WI) because of voids and Cr content in steel. Presence of Cr produces oxides during arc movement. Narrow HAZ and highest hardness at WI. Narrow HAZ is achieved due to high heat input and small welding cycle. Highest hardness is caused by high current, large amount of heat and rapid cooling Base metal contains ferrite and pearlite structure. TMAZ shows bainite formation with polygonal ferrite and has a fine grain due to high arc rotation. HAZ exhibits coarse grain structure due to low thermal impact.
ST37 tubes, 25 mm OD, 3 mm thickness [10]	Distance between the magnetising shells: 10 mm. Magnetising current: 0.1–1 A. Welding current: 100–400 A. Arc voltage: 20–27 V.	Applying the upset force, weld with different aspect than of fusion weld (FW) is achieved. Because of the thermal cycles, which are determined by the arc rotation, Windmanstaten acicular ferrite, with different grain sizes, is formed in WF. On the anode side (the right side) of the tube, there are more refined grains (Figure 13). Late upsetting is indicated by the presence of micro-pores and oxide inclusions. On the left side, pearlite reveals more diffused carbon caused by high peak temperature (Figure 14)
SG35 tubes, 76 mm OD, 16 mm thickness [11]		The HAZ was observed to be formed of complete and partial recrystallised area (Figure 15). Completely recrystallized zone has a finely distributed ferritic–pearlitic mix with grain size of 9–10 in scale and larger hardness, while the partially recrystallised zone has a structure of ferrite and pearlite with grain size of 7–8 in scale and lower hardness (181–196 HV). HAZ is the overheated area at the internal or external edges due to the arc movement on the internal or external edges, respectively, the other part maintaining the recrystallised structure appearance.
X70 steel: steering rod, 22 mm OD, 2.2 mm thickness [54]	Steering rod Welding time: 3.7 s; Upset force: 21 kN; Heat input: 6.1 kW.	Tensile and bend testing indicates high mechanical strength and high ductile properties of the joint. Metallographic tests show no defects. Pipe: HAZ contains a mix of pearlite, bainite, and a small amount of ferrite while base metal (BM) reveals ferrite and pearlite microstructure. Rod: HAZ indicates bainite–ferrite structure, while the parent metal has higher hardness. Shock absorber: HAZ and BM structure contains ferrite and pearlite, but the BM contains more ferrite. No coarse grain area was noticed.
shock-absorber, 40 mm OD, 2.2 mm thickness [54]	Shock absorber Welding time: 4.8 s; Upset force: 31 kN; Heat input: 6.7 kW.	
torque rod 34 mm OD 6.2 mm thickness [54]	Torque rod Welding time: 13.2 s; Upset force: 40 kN; Heat input: 7.2 kW.	
A213-T11, A213-T91 boiler pipes, heat exchangers [5]	Welding current: 270–290 A. Welding time: 6–9 s. Upset current: 800–1000 A. Upset time: 0.3 s.	SEM images of T11 and T91 dissimilar joint are shown in Figure 16, Figure 17 and Figure 18. The weld line shows equiaxed bainite and polygonal ferrite due to the upset pressure. TMAZ1 and TMAZ3 show the phase transformation in T11 and T91, respectively.
SA210GrA tubes, 44 mm OD, 4.5 mm thickness [44]	Upset current: 400–1200 A.	Lower upset current leads to decarburised zone and, consequently, degradation of properties. Higher upset current causes acicular ferrite (AF) formation characterised by higher strength and toughness. The thermal gradient determines three different TMAZs. Due to the rapid cooling rate, TMAZ1 has acicular ferrite and pearlite structure, with a coarse-grained aspect. This region size modifies with variation of the upset current. Due to the overheating above the recrystallization temperature, TMAZ2 shows ferrite and pearlite structure, with coarser grains compared to TMAZ3. TMAZ3 exhibits a normalised structure with fine ferrite and pearlite. BM reveals a structure of banded ferrite–pearlite.
A213-T11 tubes 47.6 mm OD, 6.6 mm thickness [45]	Welding current: 200 A. Welding time: 20 s. Upset current: 1100 A. Upset time: 0.3 s.	Appropriate depth of penetration (DOP) and uniform penetration are achieved. High UTS obtained at higher welding current and time. Small grain size, indicating good integrity and higher UTS. Grain size increases towards HAZ and BM. SEM fractography, Figure 19, shows wavy pattern indicating brittle fracture which may be caused by insufficient current and lesser welding time.
A213-T11 tubes, 47.6 mm OD, 6.6–7 mm thickness [49]	Welding current: 200–220 A. Arc heating time: 14–26 s.	No distinct HAZ and visible fusion line are observed. High instantaneous heat input and fast cooling due to short welding cycle. Radiography using X-ray shows pores in the weld reinforcement. XRD spectra show crystal phase in weld region and base metal, both with the highest peak of Cr silicate in cubical phase or Si carbide in hexagonal phase. No irregularities on the weld surface that indicate appropriate expulsion of impurities in the upsetting state. The same composition of BM and weld zone confirms no formation of inter-metallics and, therefore, no modification of post-weld material properties. The Williamson–Hall (W-H) analysis of the weldment (Figure 20) indicates increased strain in the weld region in comparison with the base metal, being caused by the shrinkage forces in the weld area. Figure 21 shows the XRD spectra of the weld region.
Low alloy steel (T11) tubes, 48 mm OD, 6 mm thickness [48]	Welding current: 270, 290 A. Upset current: 800, 1000 A.	Due to variation of cooling rates, determined by high welding current, four TMAZs were found. TMAZ1 structure comprises fine bainite and polygonal ferrite. Deformation during upsetting phase delays the growth of bainite needles. Lower hardness due to presence of ferrite and high UTS due to bainite nucleation. Delayed bainite growth was noticed for lower current. Hardness is similar to TMAZ2 since acicular ferrite is promoted by higher cooling rates and deformation. TMAZ2 exhibits upper bainite (UB) with relatively long and parallel ferrite laths. Due to the presence of UB, highest hardness was measured in this zone. TMAZ3 microstructure consists of granular bainite with equiaxed bainitic, ferritic structure and discrete martensite–austenite (MA) islands. TMAZ4 reveals recrystallised fine grain of ferrite and pearlite. Low arc current results in mechanical failure in weld. Bainite insufficient melting causes flaws in weld. SEM analysis shows dimpled structure indicating ductile fracture. Weld may contain oxides caused by atmospheric corrosion, as well as insufficient melting. BM is seen as a matrix of ferrite–pearlite.
ST 37 steel tubes 25.4 mm OD, 3 mm thickness [50]	Magnetising current: 0.1–1 A. Welding current: 100–400 A.	Macrostructure shows coarsened grains at 2 mm from the centreline, because of austenite decomposition into ferrite and pearlite. Windmanstatten ferrite and bainite structure is found in the weld zone with differing grain sizes on the two sides. Due to the difference in heat input introduced in the tubes in the arc rotation phase, the grains are finer on the anode side. Micro porosities caused by short arc rotation time and low heat input. Increased hardness in the weld and TMAZ, due to high dislocation density. Magnetic field strength that depends on the welding current density and tube gap.
TSA (tubular shaft assembly) parts: C22 steel tube, 1.5 mm thickness, and two ends: joint UC1 steel-stub shaft ended with spline [55]		Ultrasonic wave propagation was observed by using ultrasound scanner for defects detection. Collected data were processed in two ways called A-scan and B-scan that are 1-D or 2-D acoustic profiles. The wave transmission and the reflected waves were analysed in order to set appropriate parameters for the transducer. This testing method, with the transmitter orientation at 31.3 degrees, can be applied for assessment of MIAB welds. In simulation platform, failed welded samples have reflected the wave from the WI. A shorter time of wave flight indicating the lack of weld or defect occurrence. Wave reflection causes substantial differences in acoustic impedance of the weld surface and air. CT showed excess metal deposits in the mid and bottom weld, and gas pores on the surface, in accordance with the US testing results (Figure 22).
T11 and T91 Tubes OD- 44.5 mm, 4 mm thickness [56]	SEM. Vickers hardness. Potential dynamic polarisation. Radiographic testing.	Martensitic structure of 2–3 µm grain size. Hardness values (218 HV1) in weld higher than both base metals. Weld bead width of 10–12 µm and narrow HAZ. Sound joint formation as per radiographic testing.

**Table 3:** Welding parameters for automobile components.

Element	Diameter (mm)	Wall Thickness (mm)	Welding Time (s)	Upsetting Force (kN)	Part Length (mm)	Length Reduction (mm)	Consumed Power (kW)
Steering rod	22	2.2	3.7	21	300	2.1–2.3	6.1
Shock absorber	4	2.2	4.8	31	300	3.7–3.9	6.7
Torque rod	34	6.2	13.2	40	-	7.0–7.5	7.2

## Data Availability

Not applicable.
